# Prognostic significance of SLC25A28 and its associations with mitochondrial metabolism and immune features in glioma

**DOI:** 10.3389/fonc.2026.1917001

**Published:** 2026-10-30

**Authors:** Wangyang Yu, Yue Yang, Wenli Guo, Yafeng Wang, Jinsong Bai, Chen Li

**Affiliations:** Second Hospital of Hebei Medical University, Shijiazhuang, China

**Keywords:** ferroptosis, glioma, immune microenvironment, mitochondrial iron, oxidative phosphorylation, prognosis, SLC25A28

## Abstract

**Background:**

SLC25A28 encodes the mitochondrial iron transporter mitoferrin-2, but its prognostic relevance and biological associations in glioma remain incompletely characterized. We investigated its clinical significance and relationships with metabolic, ferroptosis-related and immune features across glioma cohorts.

**Methods:**

SLC25-family genes were screened in CGGA and TCGA, followed by survival analysis and meta-analysis across nine GlioVis cohorts. Clinical Cox models, prognostic scores and grade/IDH subgroups were evaluated. Metabolic, immune and curated ferroptosis-related signatures were examined using clinically adjusted models, and immune-cell and checkpoint analyses underwent false-discovery-rate correction. SLC25A28 expression was assessed by qRT-PCR and Western blotting.

**Results:**

Lower SLC25A28 expression was associated with unfavorable clinicopathological features and shorter overall survival (pooled hazard ratio, 1.91; 95% confidence interval, 1.46–2.50; I² = 84.9%). Exploratory analyses supported survival associations in both IDH-mutant and IDH-wild-type subgroups of CGGA and TCGA, although findings within glioblastoma varied. Positive oxidative-phosphorylation associations persisted after grade/IDH adjustment in both pooled cohorts; within glioblastoma, this association was supported in CGGA but not TCGA. Ferroptosis-related transcriptional associations were also detected, with consistent all-glioma WikiPathways results across sensitivity analyses in TCGA but scoring-method and cohort dependence in regulator signatures. Immune-cell differences survived correction for 10 of 22 populations in CGGA and 16 of 22 in TCGA, and three checkpoint genes showed positive correlations in both cohorts. qRT-PCR and Western blotting confirmed lower SLC25A28 expression in U87 and U251 cells than in normal human astrocytes.

**Conclusions:**

SLC25A28 is a candidate prognostic biomarker associated with mitochondrial metabolism, ferroptosis-related transcriptional features and immune features in glioma. Multi-cohort survival evidence and clinically adjusted molecular associations support further evaluation of its prognostic and biological relevance. Subtype-specific clinical validation and functional investigations will help define its clinical utility and biological role.

## 1 Introduction

Glioma is the most common primary malignant tumor of the central nervous system (1). Current standard treatment primarily consists of maximal surgical resection followed by radiotherapy and chemotherapy. However, the highly infiltrative nature of glioma cells often prevents complete surgical resection, while resistance to conventional chemoradiotherapy and the limited availability of effective targeted therapies further restrict treatment efficacy. Consequently, the prognosis of patients with glioma remains poor (2).

In 2016, the World Health Organization (WHO) classified gliomas into grades I–IV and, for the first time, incorporated molecular biomarkers into the histopathological classification system. Commonly used molecular markers include IDH1/2 mutation status, 1p/19q codeletion status, and MGMT promoter methylation status (3). The fifth edition of the WHO Classification of Tumors of the Central Nervous System (WHO CNS5), published in 2021, further emphasized the essential role of molecular diagnostics in the classification and clinical management of gliomas (4).

Gliomas are characterized by profound metabolic reprogramming. As the central hub of cellular metabolism, mitochondria play important roles in glioma biology and progression (5, 6). Integrated analyses of glioma tissues and plasma have identified shared disturbances in amino-acid metabolism and the tricarboxylic acid cycle, highlighting the potential of metabolic features for biomarker discovery (7). Metabolic exchange between mitochondria and the cytoplasm occurs across the relatively impermeable inner mitochondrial membrane and therefore relies on specialized solute carrier proteins. Dysregulation of mitochondrial transport and metabolism may consequently affect cellular energy production and metabolic homeostasis, thereby contributing to tumor progression.

The solute carrier family 25 (SLC25), also known as the mitochondrial carrier family (MCF), comprises 53 members and represents one of the largest families of mitochondrial solute transport proteins in humans. These proteins are embedded in the inner mitochondrial membrane and are essential for maintaining mitochondrial function and homeostasis (8). By mediating the transport of metabolites and other substrates across the inner mitochondrial membrane, SLC25 proteins participate in a broad range of cellular processes, including oxidative phosphorylation, amino acid metabolism, biosynthesis of iron–sulfur (Fe–S) clusters and heme, macromolecular biosynthesis, and thermogenesis (9).

Accumulating evidence indicates that SLC25 family members are involved in the development and progression of various diseases (10), and their roles in cancer are increasingly being recognized. Several SLC25 proteins have been identified as potential therapeutic targets or biomarkers in different tumor types (11). Although many SLC25 genes are widely expressed in the central nervous system (12), their clinical significance and biological roles in glioma remain insufficiently characterized.

Mitochondrial iron homeostasis is closely linked to mitochondrial function because mitochondria are major sites of cellular iron utilization, particularly for the biosynthesis of heme and iron–sulfur (Fe–S) clusters (13–15). Mitoferrins are important mitochondrial iron transporters involved in mitochondrial iron uptake and homeostasis, with Mitoferrin-1 (SLC25A37) and Mitoferrin-2 (SLC25A28) exhibiting distinct biological functions (16, 17). Importantly, previous studies have implicated mitochondrial iron metabolism in cancer biology, including glioma. For example, Mitoferrin-2 has been investigated in human glioma cells, whereas Mitoferrin-1 has been reported to promote proliferation and regulate glutathione-associated protein oxidation in glioblastoma (18, 19). These findings suggest that mitochondrial iron transport may be relevant to glioma biology. However, the prognostic significance of SLC25A28 across glioma cohorts and its potential association with ferroptosis-related processes and the tumor immune microenvironment have not been systematically characterized.

Ferroptosis is an iron-dependent form of regulated cell death characterized by the accumulation of lipid peroxidation products and has attracted increasing attention in cancer research (20, 21). In glioma, ferroptosis-related processes have been associated with tumor progression, immunosuppression, and resistance to immunotherapy (22). In experimental glioblastoma models, IRP1-mediated ferroptosis has been shown to enhance temozolomide sensitivity, linking iron homeostasis and lipid peroxidation to treatment response (23). Nevertheless, existing studies have primarily focused on ferroptosis itself or individual ferroptosis-related regulators, whereas the potential relationship between mitochondrial iron transport and ferroptosis-related phenotypes in glioma remains incompletely understood. Therefore, investigating SLC25A28 from the perspective of mitochondrial iron metabolism may provide an additional framework for understanding its potential biological and clinical significance in glioma.

In the present study, we screened SLC25-family genes in two large glioma cohorts and prioritized SLC25A28 on the basis of its prognostic association and biological relevance to mitochondrial iron transport. We integrated clinicopathological analyses, multi-cohort survival evidence, molecular and histological subgroup analyses, prognostic-score evaluation, clinically adjusted metabolic and ferroptosis-related pathway analyses, and immune profiling. Expression measurements in glioma cell lines provided complementary experimental evidence. This approach was designed to characterize the prognostic relevance of SLC25A28 and the metabolic, ferroptosis-related and immune contexts associated with its expression.

## 2 Materials and methods

### 2.1 Data sources and study populations

Transcriptomic and clinical data were obtained through GlioVis (24). CGGA and TCGA-GBMLGG, hereafter TCGA, were used for gene screening and detailed clinical and molecular analyses. Survival associations were evaluated in nine datasets: CGGA, TCGA, Freije, Gravendeel, Kamoun, LeeY, Murat, Phillips and Rembrandt. Normalized, transformed expression values supplied by the portal were analyzed on their existing scales. Cohort characteristics and analysis-specific sample accounting are provided in **Supplementary Tables S1**, **S2**.

The source populations comprised 1,018 CGGA and 669 TCGA records with matched expression and clinical annotations. Survival analyses required recorded follow-up and event status; expression-based survival plots included 983 CGGA and 667 TCGA patients. Clinical models used complete cases for the variables being analyzed, without imputing missing clinical values. Sample sizes therefore differed between analyses. Tumor grade, histology, IDH status and other molecular features were defined from the source annotations; historical GBM designations were retained for subgroup analyses.

The SLC25 family comprised 53 genes based on the published family definition (8). The two primary cohorts were analyzed separately, and the agreement and variation of their results were considered together.

### 2.2 Screening and prioritization of SLC25-family genes

Continuous expression of each SLC25 gene was evaluated using univariable Cox regression for overall survival (OS). Genes passing a Benjamini–Hochberg (BH)-adjusted P< 0.01 threshold were evaluated in cohort-specific SLC25-family multivariable models, with P< 0.01 used to identify retained candidates. These family models evaluated associations conditional on the included genes; clinical covariates were assessed in separate models.

Candidates significant in both CGGA and TCGA were considered for further study. SLC25A28 was prioritized because mitoferrin-2 participates in mitochondrial iron transport and has been implicated in oxidative injury in glioma cells (16, 18). Comparative results for SLC25A28, SLC25A1, SLC25A39 and SLC25A37 are reported in **Supplementary Table S3**.

### 2.3 Clinicopathological and clinical survival analyses

SLC25A28 expression was compared across WHO grade, IDH status, 1p/19q codeletion status and MGMT promoter methylation categories. Pairwise comparisons used Wilcoxon rank-sum tests. Clinical Cox models evaluated continuous SLC25A28 expression together with available clinical and molecular variables. Clinical covariates were entered using the coding of the cohort-specific model; the reported hazard ratios correspond to the displayed model terms.

The CGGA clinical analyses considered age, sex, WHO grade, IDH status, 1p/19q codeletion, primary/recurrent/secondary tumor status, radiotherapy, chemotherapy and MGMT promoter methylation. The TCGA clinical model included expression, age, sex, grade, IDH and 1p/19q status. Schoenfeld residuals were used to assess proportional hazards. Coefficients from models containing variables with non-proportional effects were interpreted as model-conditional associations.

### 2.4 Cohort and subgroup survival analyses

For the primary Kaplan–Meier analyses, patients were divided using the median SLC25A28 expression within each cohort. The CGGA and TCGA median expression cutoffs were 5.49 and 9.46, respectively, on their supplied expression scales. Survival differences were evaluated using two-sided log-rank tests, and hazard ratios (HRs) compare low with high expression. Continuous expression was retained in the gene-screening and clinical Cox models.

Exploratory analyses evaluated IDH-mutant, IDH-wild-type, historical GBM, GBM/IDH-mutant and GBM/IDH-wild-type tumors separately in CGGA and TCGA. These subgroup plots used the GlioVis optimal-cutpoint function. Because the cutpoints were selected using survival outcomes, subgroup estimates and nominal P values were interpreted as exploratory. Exact group sizes and cutpoints are provided in **Supplementary Table S2**.

### 2.5 Meta-analysis and cohort heterogeneity

Cohort-specific log HRs and standard errors from the nine median-split analyses were combined using a random-effects meta-analysis. Heterogeneity was summarized using I², and leave-one-out analyses assessed the influence of individual datasets on the pooled estimate and heterogeneity. Cohorts were included irrespective of the direction or statistical significance of their associations.

An exploratory cohort-composition analysis compared GBM-only datasets (LeeY and Murat) with seven mixed-glioma datasets, classified using GlioVis descriptions. Subgroup estimates were calculated by restricted maximum likelihood (REML). The composition contrast was evaluated using mixed-effects meta-regression with a common residual heterogeneity variance and modified Hartung–Knapp inference, with the variance multiplier constrained to be at least one. This *post hoc* dataset-level comparison was interpreted cautiously because only two cohorts were GBM-only. A separate optimal-cutpoint meta-analysis and its leave-one-out results were treated as sensitivity analyses. Dataset identifiers, effect estimates and computational details are provided in **Supplementary Table S4** and the **Supplementary Methods**.

### 2.6 Prognostic modeling and score evaluation

SLC25A28 expression, age, IDH status and WHO grade were identified as shared clinical-model factors in CGGA and TCGA and were considered as candidate inputs for prognostic modeling. LASSO-Cox regression was performed in CGGA using glmnet, with the penalty selected by 10-fold cross-validation. Patient risk scores were calculated as the weighted sum of predictor values and fitted coefficients:


$$\text{Risk Score}=\overset{n}{\underset{i=1}{\sum }}({\text{Coef}}_{i}\times X_{i})$$

Here, Coef denotes a fitted regression coefficient and X the corresponding predictor value.

A clinical nomogram incorporating SLC25A28 expression, age, IDH status and WHO grade was constructed using rms and hdnom to estimate 3-year OS. Whole-cohort survival stratification used the CGGA-derived four-variable score: 0.00419 × age − 0.05722 × SLC25A28 expression − 0.46846 × IDH code + 0.8419 × grade code. IDH-wild-type and mutant status were coded 1 and 2, and WHO grades II, III and IV were coded 1, 2 and 3. Kaplan–Meier analyses used the median score within each cohort. TCGA provided cross-cohort evaluation; it also contributed to the upstream selection of candidate variables.

Time-dependent receiver operating characteristic (ROC) curves and corresponding areas under the curve (AUCs) at 1, 3 and 5 years were estimated for the continuous CGGA-derived four-variable risk score in each cohort using the Kaplan–Meier method implemented in the survivalROC package, with overall survival converted from months to years.

A separate CGGA score containing SLC25A28 expression and age was applied within six grade-by-IDH groups and within the IDH-mutant/WHO grade II–III population. Each subgroup used its own risk-score median, with the score coefficients held fixed. Schoenfeld-residual diagnostics were evaluated for the expression-and-age model. Calibration at 1, 3 and 5 years was assessed in the CGGA development sample. Exploratory decision curves evaluated the individual predictors SLC25A28, age, IDH and grade against treat-all and treat-none strategies; these were distinct from evaluation of the combined nomogram.

### 2.7 Metabolic and immune pathway scoring and clinical adjustment

Gene set variation analysis (GSVA) was used to characterize metabolic and immune transcriptional programs (25). Eight biologically selected signatures from MSigDB human release 2023.1 were evaluated: KEGG oxidative phosphorylation; GO hypoxia-inducible factor 1alpha signaling and adaptive immune response; and Hallmark oxidative phosphorylation, hypoxia, inflammatory response, interferon alpha response and interferon gamma response. Scores were calculated separately within each cohort using a Gaussian kernel and then held fixed for subgroup analyses. Constant genes were excluded, and SLC25A28 was removed from gene sets to avoid direct self-inclusion. Matched gene coverage is reported in **Supplementary Table S6**.

Linear models related each pathway score to continuous SLC25A28 expression. Predictor and outcome were standardized within the analytic population, and unadjusted and adjusted estimates were compared on identical samples. Primary models included categorical WHO grade and IDH status. Extended models additionally included age, sex and, for CGGA, primary/recurrent/secondary tumor status. Analyses were performed in the full cohorts, individual grade and IDH strata, historical GBM, and GBM/IDH-wild-type tumors. Covariates constant within a subgroup were omitted, and complete-case groups with fewer than 30 patients were not modeled.

Estimates were reported with HC3 heteroskedasticity-robust 95% confidence intervals. BH correction covered the 16 cohort–signature tests within each model or stratum. Sensitivity analyses evaluated shared gene coverage, expression-score definitions, additional 1p/19q adjustment, primary-tumor restriction, rank and robust regression, extreme observations and influential cases. Restricted cubic splines with knots at the 5th, 35th, 65th and 95th expression percentiles were used to examine nonlinearity in GBM and GBM/IDH-wild-type tumors. The **Supplementary Methods** and **Supplementary Tables S7**, **S8** provide full specifications, testing families and sensitivity results.

### 2.8 Ferroptosis-related transcriptional analyses

Ferroptosis-related transcriptional features were evaluated using the WikiPathways ferroptosis set from MSigDB 2026.1.Hs (WP_FERROPTOSIS) (26) and separate FerrDb V3 driver and suppressor sets (27), downloaded on October 4, 2026. FerrDb annotations were restricted to ferroptosis, human-organism annotations and protein-coding genes with verified identities. Genes assigned both driver and suppressor roles were excluded from the directional sets. Primary sets retained all eligible confidence categories; a sensitivity analysis retained only genes with an eligible annotation rated Validated. Following HGNC harmonization and averaging of duplicate features, both cohorts used the same 16,659 variable genes as background and identical matched sets of 63 WikiPathways genes, 300 drivers and 549 suppressors. SLC25A28 was excluded from all scoring sets and the background. GSVA scores were calculated separately within each full cohort using a Gaussian kernel, weight 1, mx_diff = True and abs_rnk = False, and held fixed for subgroup analyses (**Supplementary Table S9**).

Continuous SLC25A28 expression and signature scores were standardized within each complete-case population. Linear models used the grade/IDH and extended adjustments described above, with HC3 robust, t-based confidence intervals. The primary BH correction covered six signature–cohort tests. Analyses included IDH-defined groups, historical GBM, GBM/IDH-wild-type and tumors meeting GBM/WHO grade IV/IDH-wild-type criteria. Sensitivity analyses evaluated mean gene-wise z scores, rank transformation, exclusion of extreme expression values, primary tumors, matched-sample 1p/19q adjustment and Validated-evidence sets. Restricted cubic splines used knots at the 5th, 35th, 65th and 95th expression percentiles. Exploratory gene-level analyses included all 63 matched WikiPathways genes. *Post hoc* descriptive checks examined global expression distributions following discordant sensitivity estimates. Complete specifications and testing families are provided in the **Supplementary Methods** and **Supplementary Tables S9**–**S11**. These scores characterize transcriptional features rather than measured ferroptosis activity.

### 2.9 Immune-cell and immune-checkpoint analyses

The Immune Estimation module of TIMER2.0 was used to obtain CIBERSORT estimates of 22 relative immune-cell fractions. The expression-group annotations accompanying the CIBERSORT data were used for comparisons (CGGA: 509 high and 509 low; TCGA: 335 high and 334 low). Differences were tested using the Kruskal–Wallis test with correction for tied ranks. Group definitions and sample accounting are documented in the **Supplementary Methods**.

Two-sided Pearson correlations related continuous SLC25A28 expression to PDCD1, CD274, CTLA4, HAVCR2, LAG3, TIGIT, VTCN1, C10orf54, BTLA and ICOS on the supplied normalized, transformed expression scales. BH adjustment was performed separately for the 22 cell-fraction comparisons and the ten checkpoint correlations within each cohort. Adjusted P< 0.05 was used to identify associations, and all nominal and adjusted values are reported in **Supplementary Table S5**.

### 2.10 Experimental validation

#### 2.10.1 Cell lines and cell culture

Human glioma cell lines U251 and U87 were kindly provided by the Department of Pathology of the authors’ institution. Normal human astrocytes (NHA) were purchased from Guangzhou Jennio Biotech Co., Ltd.

Cells were cultured in high-glucose Dulbecco’s modified Eagle’s medium (DMEM) supplemented with 10% fetal bovine serum (FBS) and 1% penicillin–streptomycin. Cells were maintained in a humidified incubator at 37 °C with 5% CO_2_.

#### 2.10.2 RNA extraction and quantitative real-time PCR

Total RNA was extracted from cultured cells using a commercial RNA extraction kit according to the manufacturer’s instructions. The extracted RNA was stored at −80 °C until further analysis.

Reverse transcription was performed using a commercial RNA reverse transcription kit according to the manufacturer’s instructions. Quantitative real-time PCR (qRT-PCR) was performed using SYBR Green I chemistry on an Mx3005P real-time PCR system (Stratagene, USA).

The primer sequences were as follows:

GAPDH
Forward: 5′-GGACGGAGATCCCCTCCAAAAT-3′
Reverse: 5′-GGCTGTTGTCATACTTCTCATGG-3′
SLC25A28
Forward: 5′-CTGGAGCACTGCGTGATGTA-3′
Reverse: 5′-AGAGGGCCTCCAACACATTG-3′

The qRT-PCR reaction volume was 20 μL and contained 4 μL of 5× Fast SYBR Green qPCR Mix, 0.2 μL of forward primer, 0.2 μL of reverse primer, 2 μL of cDNA template, and 13.6 μL of nuclease-free water.

The amplification protocol consisted of an initial denaturation at 95 °C for 30 s, followed by 40 cycles of denaturation at 95 °C for 5–10 s and annealing/extension at 60 °C for 20 s.

GAPDH was used as the endogenous reference gene. Relative SLC25A28 expression was calculated using the comparative threshold cycle method (2^−ΔΔCt^). Each experiment was independently repeated three times. Statistical analyses and graphical presentation were performed using GraphPad Prism 9. Differences among groups were assessed using one-way analysis of variance (ANOVA), and P< 0.05 was considered statistically significant.

#### 2.10.3 Western blot analysis

Cells were harvested when they reached approximately 80% confluence. Following routine digestion and centrifugation, cells were washed with phosphate-buffered saline (PBS) and collected by centrifugation at 3,000 rpm for 2 min at 4 °C.

Cell pellets were lysed in RIPA lysis buffer supplemented with a protease inhibitor cocktail and phosphatase inhibitors. The final concentrations of the protease inhibitor cocktail and phosphatase inhibitor were 1:100 and 1:10, respectively. Cell lysates were incubated on ice for 1 h with intermittent mixing and subsequently centrifuged at 13,000 rpm for 20 min at 4 °C. The supernatants were collected and stored at −80 °C until further analysis.

Protein concentrations were determined using a bicinchoninic acid (BCA) protein assay according to the manufacturer’s instructions. Equal amounts of protein were mixed with 6× protein loading buffer and denatured at 95 °C for 10 min.

Proteins were separated by sodium dodecyl sulfate–polyacrylamide gel electrophoresis (SDS-PAGE) and electrophoretically transferred onto polyvinylidene difluoride (PVDF) membranes. PVDF membranes were activated in methanol before transfer. Following transfer, membranes were washed with Tris-buffered saline containing 0.1% Tween-20 (TBST) and blocked with 5% skimmed milk in TBST for 2 h at room temperature.

Membranes were incubated overnight at 4 °C with primary antibodies against SLC25A28 and β-actin, followed by incubation with an HRP-conjugated goat anti-rabbit secondary antibody for 2 h at room temperature. The primary and secondary antibodies were diluted at 1:1,000 and 1:2,000, respectively.

Protein bands were visualized using an enhanced chemiluminescence (ECL) detection system and imaged using an Odyssey imaging system. β-actin was used as the loading control. Band intensities were quantified using ImageJ software. Each experiment was independently repeated three times. Statistical analyses were performed using GraphPad Prism 9, and differences among groups were assessed using one-way ANOVA. P< 0.05 was considered statistically significant.

### 2.11 Statistical analysis

All tests were two-sided. Cox regression and survival analyses used R 4.3.1, including survival 3.5.7, meta 6.5.0, glmnet, rms 6.7.1 and hdnom 6.2.1. Pathway scoring and adjusted models used Python 3.12.14, GSEApy 1.3.1 and statsmodels 0.15.0. P< 0.05 was used for individual tests unless a different screening or multiple-testing threshold was specified. BH-adjusted P values are denoted q values. Effect sizes, confidence intervals and consistency across cohorts were considered alongside statistical significance.

## 3 Results

### 3.1 SLC25A28 emerges from SLC25-family screening

The family screen identified SLC25A28 and SLC25A1 as candidates associated with OS in both CGGA and TCGA (**Figure 1**). In the SLC25-family multivariable models, SLC25A28 had HRs of 0.65 (95% CI, 0.53–0.80; P< 0.001) in CGGA and 0.47 (0.29–0.76; P = 0.0021) in TCGA. The corresponding SLC25A1 estimates were 0.75 (0.64–0.88; P< 0.001) and 0.61 (0.45–0.83; P = 0.0018). SLC25A39 was retained in CGGA but not TCGA, while SLC25A37 was retained in TCGA but not CGGA. These comparisons, together with the role of mitoferrin-2 in mitochondrial iron transport, supported further investigation of SLC25A28 (**Supplementary Table S3**).

**Figure 1:**
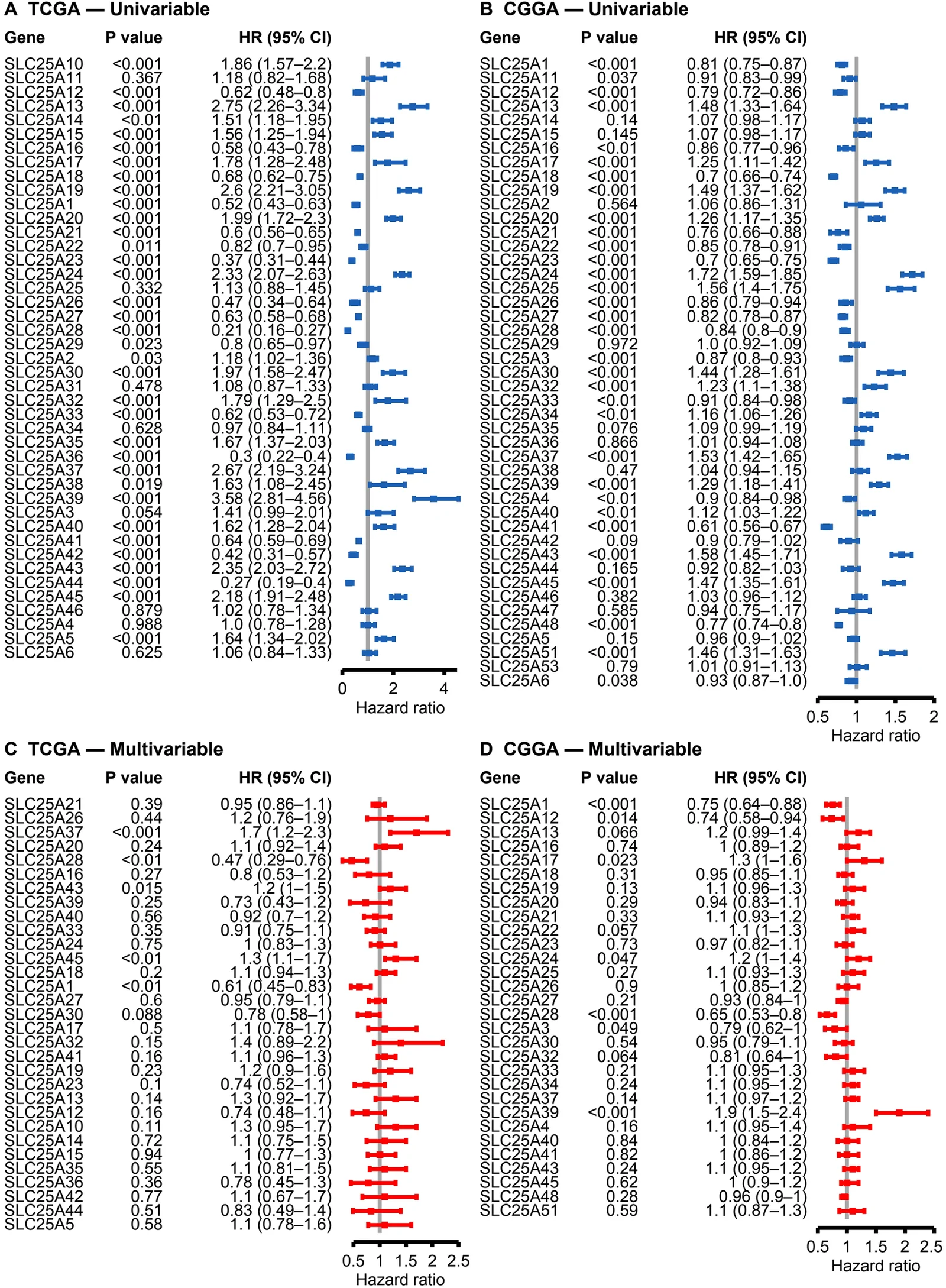
Cohort-specific Cox regression analyses of SLC25-family genes. Univariable analyses are shown for TCGA **(A)** and CGGA **(B)**, and SLC25-family multivariable models for TCGA **(C)** and CGGA **(D)**. Points indicate hazard ratios and horizontal bars 95% confidence intervals; the vertical line marks a hazard ratio of 1. P values are shown alongside the estimates. Family multivariable models assess associations conditional on the included genes and are separate from the clinical models.

### 3.2 Lower expression accompanies adverse clinicopathological features

SLC25A28 expression was associated with clinical and molecular characteristics in both primary cohorts (**Figure 2**). Lower expression occurred in higher-grade tumors and in tumors with IDH-wild-type status, 1p/19q non-codeletion or an unmethylated MGMT promoter. These relationships connect SLC25A28 expression with established glioma characteristics and support evaluating survival and pathway associations within more specific disease groups.

**Figure 2:**
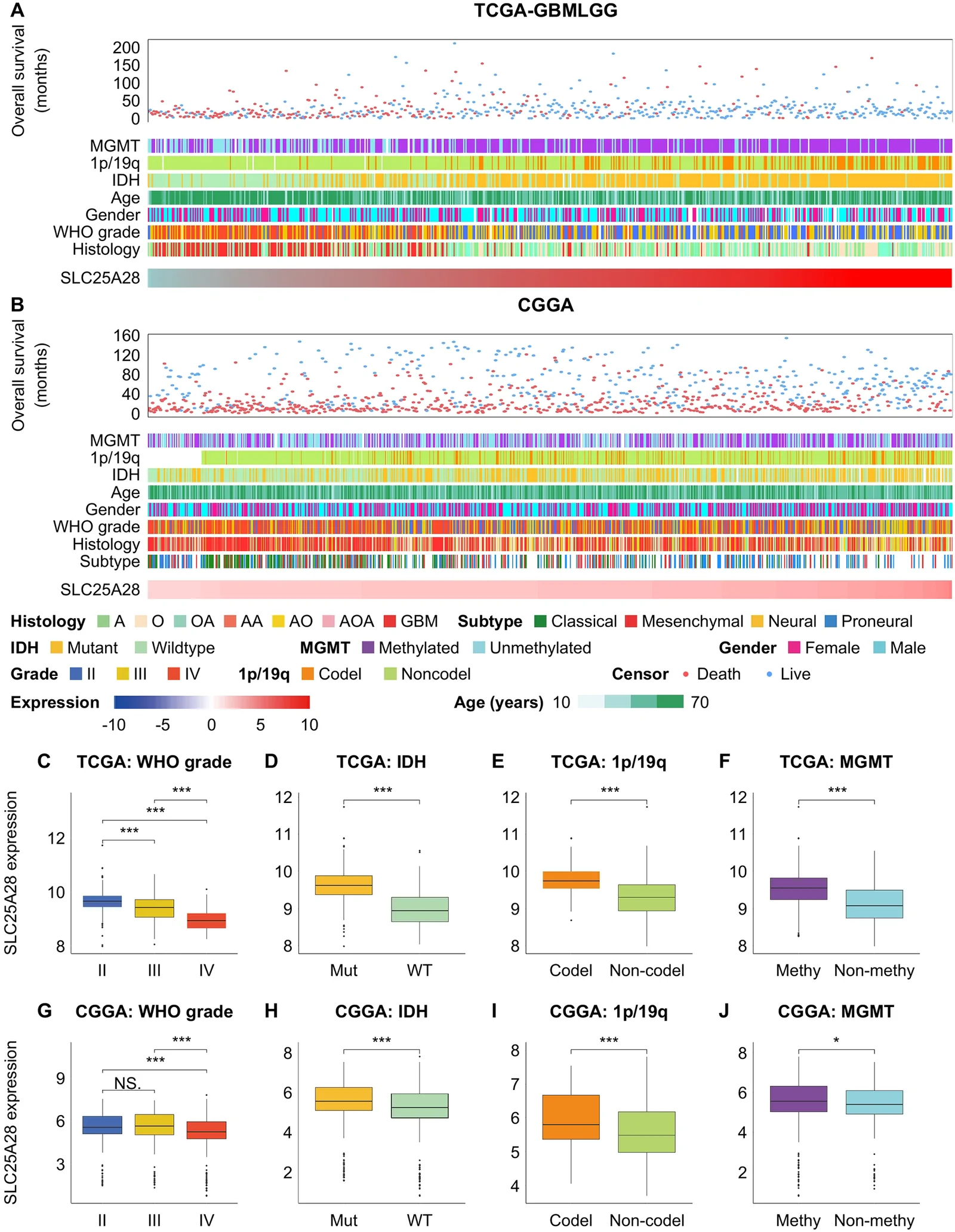
SLC25A28 expression and clinicopathological characteristics. Expression, survival and clinical annotations are shown for TCGA **(A)** and CGGA **(B)**. Box plots compare expression by WHO grade **(C, G)**, IDH status **(D, H)**, 1p/19q codeletion **(E, I)** and MGMT promoter methylation **(F, J)**; **(C–F)** show TCGA and **(G–J)** show CGGA. Boxes indicate the interquartile range, central lines the median, whiskers values within 1.5 times the interquartile range, and dots outliers. Pairwise Wilcoxon rank-sum tests: *P<0.05, **P<0.01, ***P<0.001; NS, P≥0.05.

### 3.3 Multi-cohort survival evidence and molecular subgroups

In the median-split analyses, low SLC25A28 expression was associated with shorter OS in CGGA (HR, 1.49; 95% CI, 1.26–1.75), TCGA (3.43; 2.56–4.61), Rembrandt, Gravendeel, Phillips, Kamoun and Freije. Individual estimates in LeeY and Murat were not statistically significant. The nine-cohort random-effects analysis supported an overall association with poorer survival (HR, 1.91; 95% CI, 1.46–2.50; **Figure 3**). Leave-one-out estimates remained above one, with HRs ranging from 1.75 to 2.12 (**Supplementary Figure S1**).

**Figure 3:**
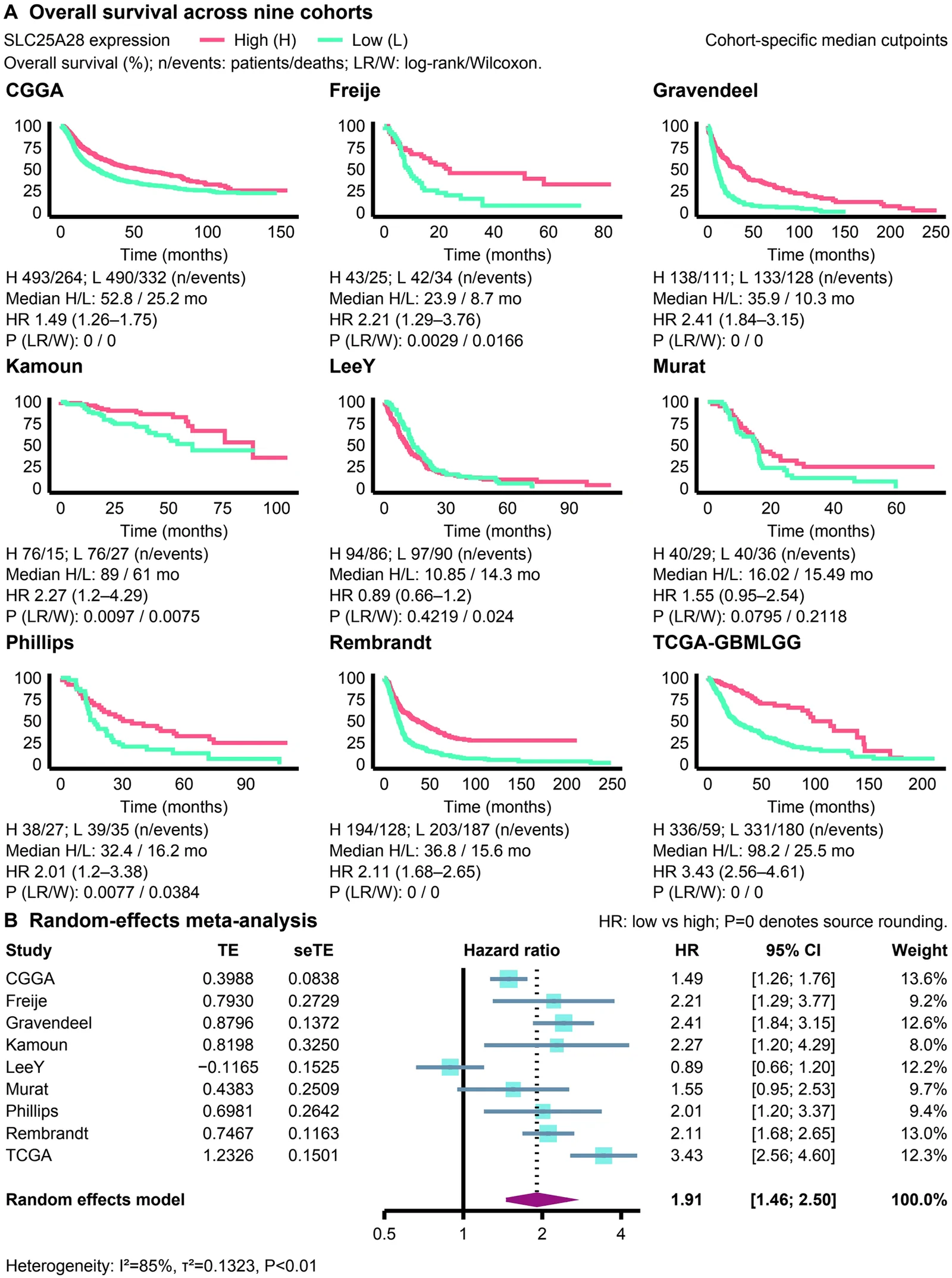
SLC25A28 and overall survival across nine glioma cohorts. **(A)** Kaplan–Meier curves use each cohort’s median expression; cohorts are arranged as CGGA, Freije, Gravendeel, Kamoun, LeeY, Murat, Phillips, Rembrandt and TCGA-GBMLGG. Pink and green denote high (H) and low (L) expression. Below each panel, n/events denotes patients/deaths; median survival is in months; HRs compare low with high expression with 95% confidence intervals; LR/W denotes log-rank/Wilcoxon P values. P values displayed as zero reflect rounding in the source analyses. **(B)** Random-effects meta-analysis. Squares show cohort estimates, horizontal bars 95% confidence intervals and the diamond the pooled estimate. TE denotes log(HR), seTE its standard error and weight the random-effects weight. I² is rounded in the plot.

Exploratory subgroup analyses supported associations in both IDH-defined groups (**Figure 4**). In IDH-wild-type tumors, low-expression HRs were 1.42 (95% CI, 1.12–1.82) in CGGA and 1.60 (1.11–2.31) in TCGA; in IDH-mutant tumors, the corresponding estimates were 1.87 (1.42–2.47) and 3.70 (2.24–6.11). Within historical GBM, the estimates were 1.30 (1.04–1.63) in CGGA and 1.37 (0.75–2.50) in TCGA. Neither cohort showed a nominally significant association in the GBM/IDH-wild-type subset. Thus, the IDH-stratified findings support the broader prognostic association, while GBM-restricted results indicate variation across disease settings.

**Figure 4:**
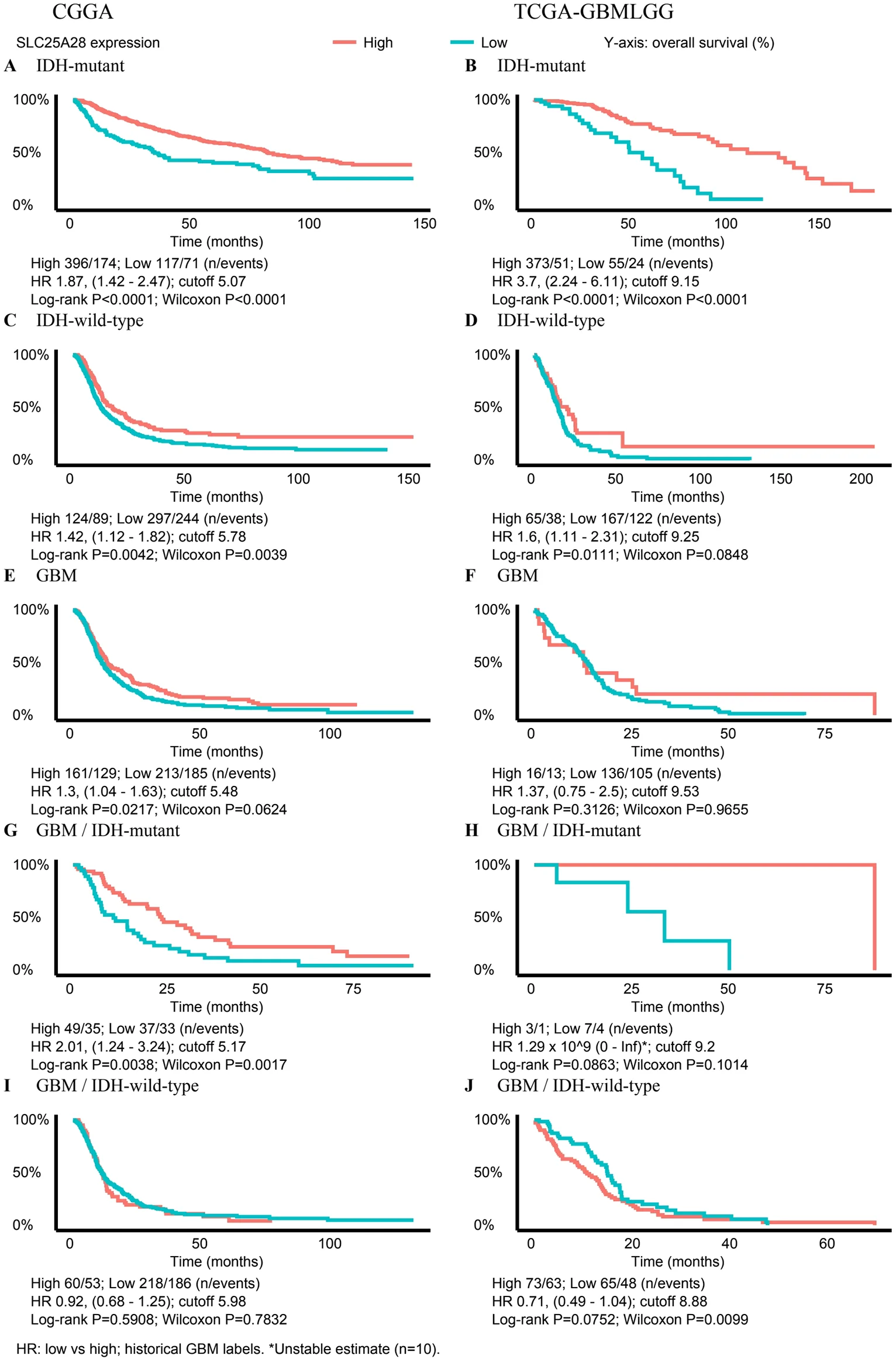
Expression-based survival associations within glioma subgroups. CGGA appears on the left and TCGA on the right. Rows show IDH-mutant **(A, B)**, IDH-wild-type **(C, D)**, historical GBM **(E, F)**, GBM/IDH-mutant **(G, H)** and GBM/IDH-wild-type **(I, J)** tumors. Red and cyan indicate high and low expression. Hazard ratios compare low with high expression; group sizes, events, 95% confidence intervals and test results are shown. Subgroup-specific cutpoints were selected using survival outcomes, so estimates and nominal P values are exploratory. The TCGA GBM/IDH-mutant group contains ten patients. Historical diagnoses follow source annotations.

Between-cohort heterogeneity was substantial in the primary meta-analysis (I² = 84.9%) and persisted after sequential exclusion of individual cohorts (I² = 75.5–86.7%). In the exploratory composition analysis, the seven mixed-glioma datasets had a pooled HR of 2.19 (95% CI, 1.74–2.76; I² = 78.3%), whereas the two GBM-only datasets had an HR of 1.13 (0.66–1.94; I² = 72.0%; **Figure 5**). The modified Hartung–Knapp moderator test yielded P = 0.0354. The small-sample-adjusted interval for the two-cohort GBM-only estimate was wide (0.034–37.35), indicating limited precision.

**Figure 5:**
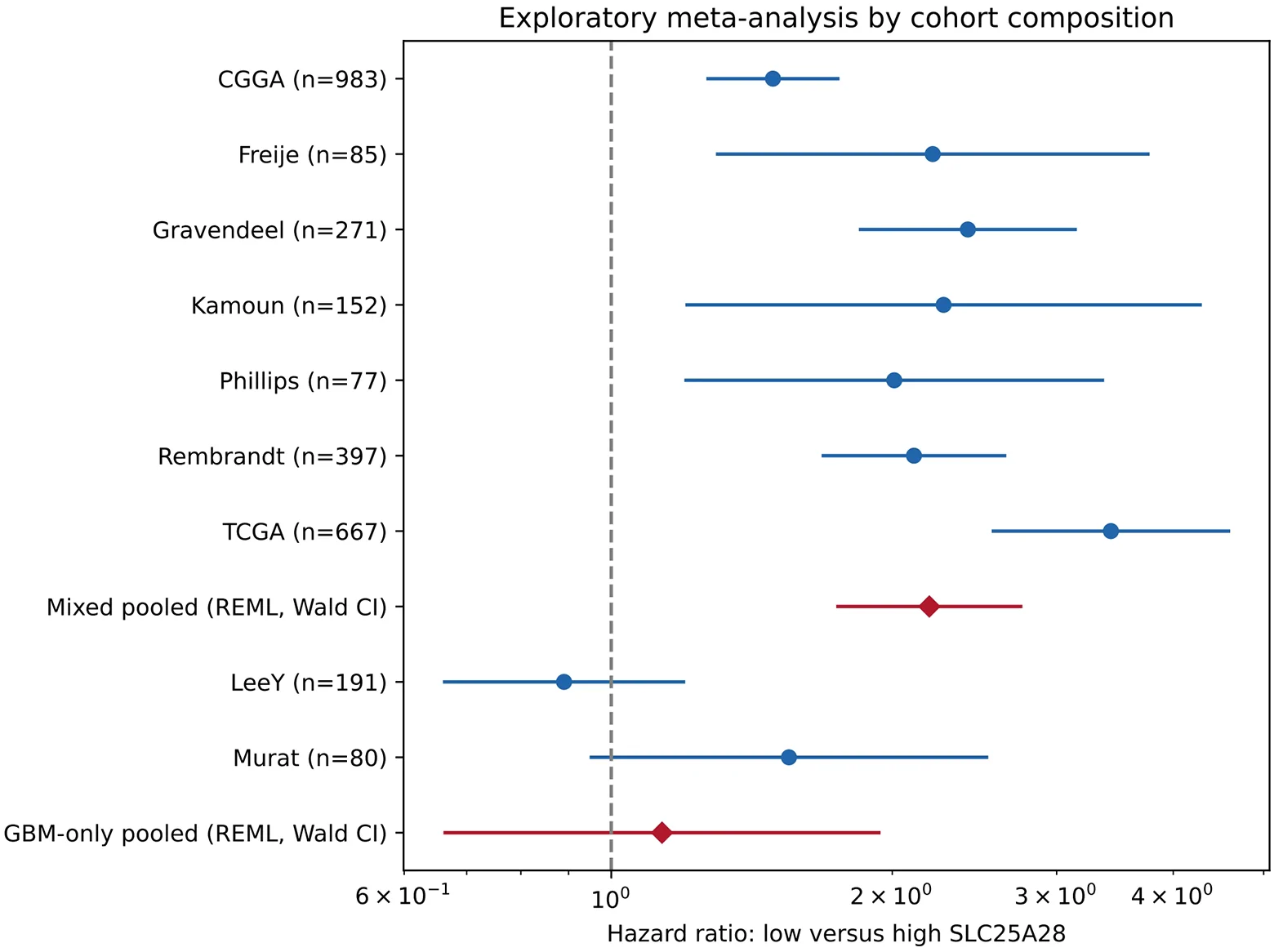
Survival associations stratified by cohort composition. LeeY and Murat were classified as GBM-only datasets and the remaining seven as mixed glioma datasets. Hazard ratios compare low with high expression using cohort-specific median cutpoints. Diamonds show REML subgroup estimates with conventional Wald 95% confidence intervals. The composition contrast was assessed using mixed-effects meta-regression and modified Hartung–Knapp inference (P = 0.0354). This exploratory dataset-level classification includes only two GBM-only cohorts and may differ from the exact composition of individual survival-analysis subsets.

The separate optimal-cutpoint sensitivity analysis yielded a pooled HR of 2.62 (95% CI, 2.06–3.34; I² = 70.0%). Excluding TCGA from that analysis yielded an HR of 2.37 (2.04–2.76) and I² = 28.6% (**Supplementary Figure S2**). The direction of the pooled association was preserved across these analyses, while the effect size and heterogeneity varied with cohort composition and cutpoint strategy (**Supplementary Table S4**).

### 3.4 Clinical prognostic associations and risk stratification

Clinical Cox analyses identified SLC25A28 expression, age, IDH status and WHO grade as factors associated with OS in both CGGA and TCGA (**Figure 6**). SLC25A28 remained associated with survival after adjustment for the included covariates, supporting its prognostic relevance within these model specifications.

**Figure 6:**
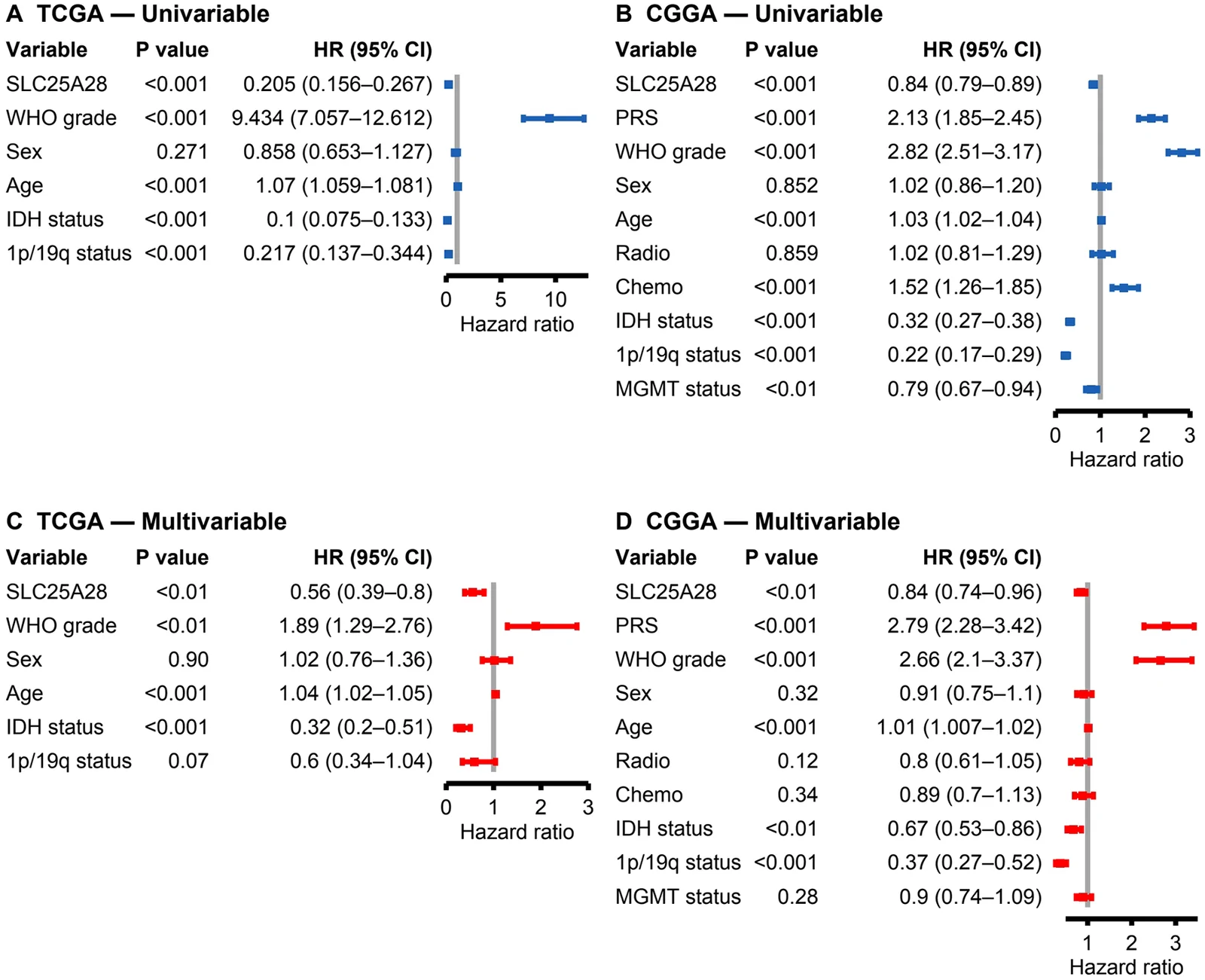
Clinical Cox regression analyses of overall survival. Univariable analyses are shown for TCGA **(A)** and CGGA **(B)**, and clinical multivariable analyses for TCGA **(C)** and CGGA **(D)**. The variables displayed in each panel were evaluated using the model coding described in the Methods. Points indicate hazard ratios and bars 95% confidence intervals; the vertical reference line marks a hazard ratio of 1. Blue and red distinguish univariable and multivariable estimates. PRS denotes primary, recurrent or secondary tumor status; Radio, radiotherapy; Chemo, chemotherapy; 1p/19q status, 1p/19q codeletion; and MGMT status, MGMT promoter methylation. Displayed P values refer to the corresponding model coefficients.

A CGGA clinical nomogram incorporating SLC25A28 expression, age, IDH status and WHO grade provided estimates of 3-year survival probability (**Figure 7**).

**Figure 7:**
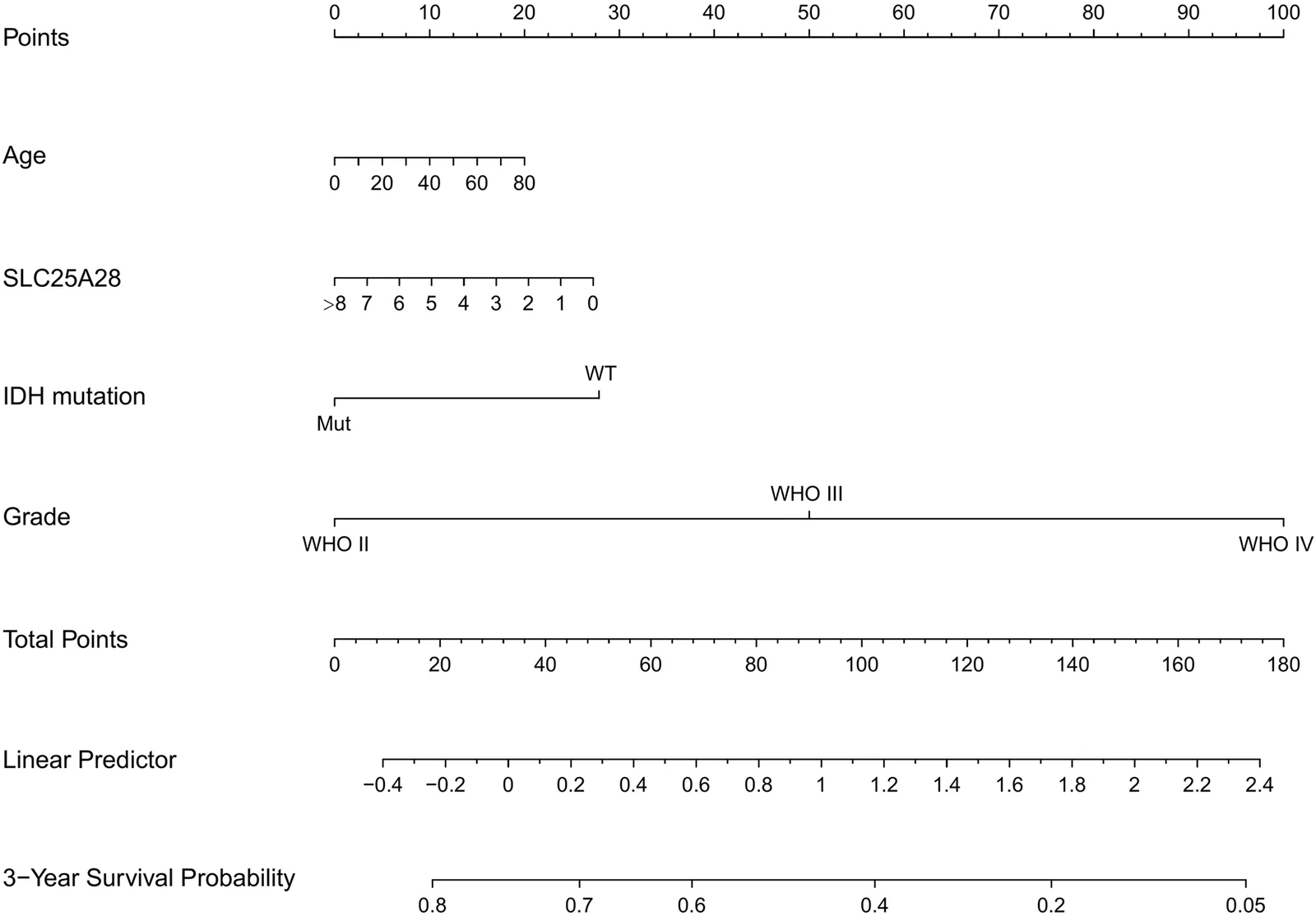
Nomogram for estimated 3-year overall survival based on the CGGA model. Age, SLC25A28 expression, IDH status and WHO grade are converted to points; their sum is mapped to the total-points, linear-predictor and 3-year survival-probability scales. Mut and WT denote IDH-mutant and IDH-wild-type status.

Risk-score groups showed separation of OS in CGGA and TCGA (both log-rank P< 0.0001; **Figures 8A, B**). In the 415-patient CGGA IDH-mutant/WHO grade II–III population, application of the SLC25A28-plus-age score separated 211 low-risk and 204 high-risk patients (log-rank P = 0.00015; **Figure 8C**). Across the six grade-by-IDH groups, separation was observed in IDH-mutant grades II and III and IDH-wild-type grade II, with nominal P values of 0.016, 0.009 and 0.040, respectively; the remaining groups did not show nominal significance (**Supplementary Figure S3**).

**Figure 8:**
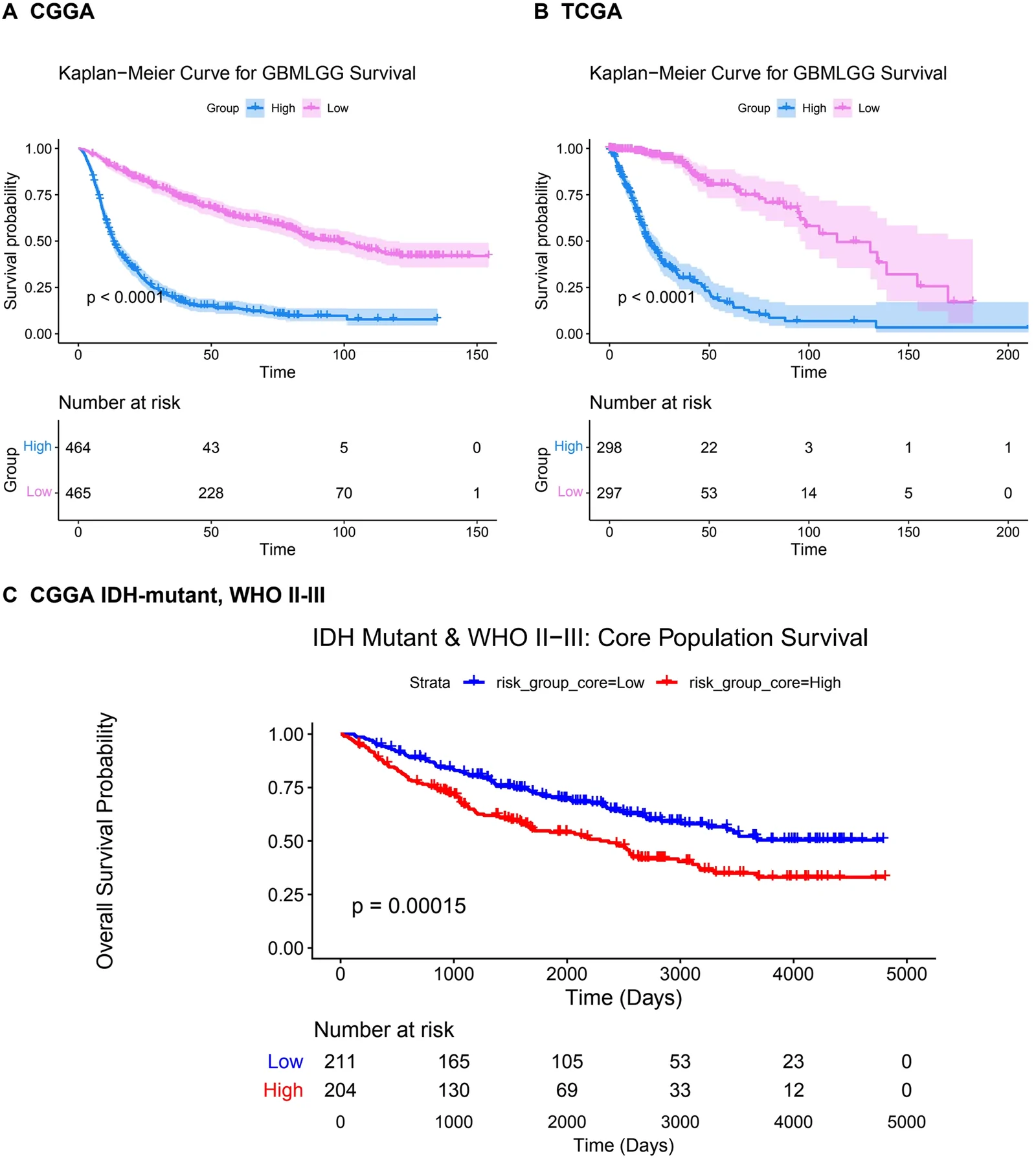
Risk-score stratification of overall survival. Kaplan–Meier curves show CGGA [**(A)**; n=929], TCGA [**(B)**; n=595] and the CGGA IDH-mutant/WHO grade II–III subgroup [(C); n=415]. A/B apply the CGGA-derived four-variable score containing SLC25A28, age, IDH and grade. **(C)** applies the separate SLC25A28-plus-age score with fixed coefficients (211 low-risk, 204 high-risk). Groups use the median score within the displayed population. **(A, B)** use blue for high risk and pink for low risk; **(C)** uses blue for low risk and red for high risk. Ticks denote censoring, and numbers at risk appear below each plot. Time is shown in months in **(A, B)** and days in **(C)**. P values are from log-rank tests.

Schoenfeld-residual tests for the SLC25A28-plus-age model yielded P = 0.7069 for expression, P = 0.1581 for age and a global P = 0.3333 (**Supplementary Figure S4**). Development-sample calibration at 1, 3 and 5 years showed agreement over some prediction ranges, with deviations in others (**Supplementary Figure S5**). Individual-predictor decision curves showed threshold-dependent net benefit without a consistent advantage over the reference strategies (**Supplementary Figure S6**).

The CGGA-derived risk score yielded time-dependent AUCs of 0.764, 0.813 and 0.799 at 1, 3 and 5 years, respectively, in CGGA (**Figure 9A**). Application of the same scoring coefficients to TCGA yielded corresponding AUCs of 0.860, 0.907 and 0.867, providing complementary evidence of discrimination in the cross-cohort evaluation (**Figure 9B**).

**Figure 9:**
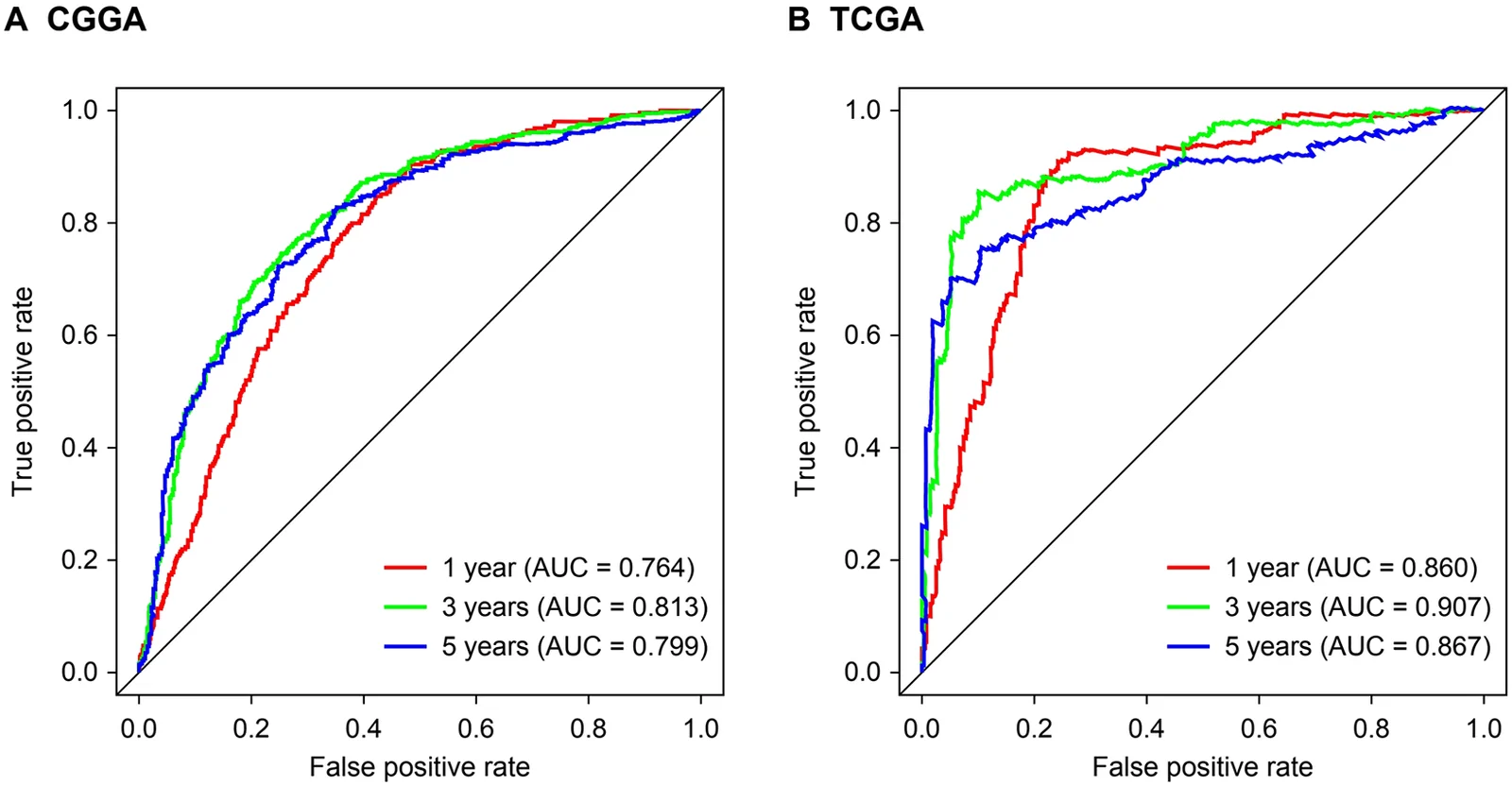
Time-dependent discrimination of the CGGA-derived prognostic risk score. ROC curves at 1, 3 and 5 years are shown for CGGA **(A)** and TCGA **(B)**, using the same CGGA-derived four-variable scoring coefficients. Red, green and blue curves represent the 1-, 3- and 5-year assessments, respectively; diagonal lines indicate chance-level discrimination. AUCs were 0.764, 0.813 and 0.799 in CGGA and 0.860, 0.907 and 0.867 in TCGA, respectively. Curves were estimated using the Kaplan–Meier method in survivalROC. TCGA results represent cross-cohort evaluation because TCGA also contributed to upstream candidate selection.

### 3.5 SLC25A28 is associated with oxidative-phosphorylation programs

Grade/IDH-adjusted analyses included 961 CGGA and 615 TCGA patients. SLC25A28 expression was positively associated with KEGG oxidative-phosphorylation scores in both cohorts, with standardized coefficients of 0.617 (95% CI, 0.573–0.661) and 0.481 (0.390–0.571), respectively (both q< 0.001). Extended adjustment yielded coefficients of 0.598 (0.553–0.644; n = 960) and 0.458 (0.366–0.551; n = 596). These findings indicate that measured grade and IDH composition did not fully account for the pooled metabolic association.

Within historical GBM, extended-adjusted coefficients were 0.577 (95% CI, 0.505–0.649; n = 378) in CGGA and 0.058 (−0.115–0.231; n = 148) in TCGA. Restriction to GBM/IDH-wild-type tumors yielded coefficients of 0.578 (0.496–0.660; n = 288) and 0.064 (−0.116–0.244; n = 138), respectively (**Figure 10**). The same cohort distinction persisted after gene-symbol harmonization and restriction to a shared gene background.

**Figure 10:**
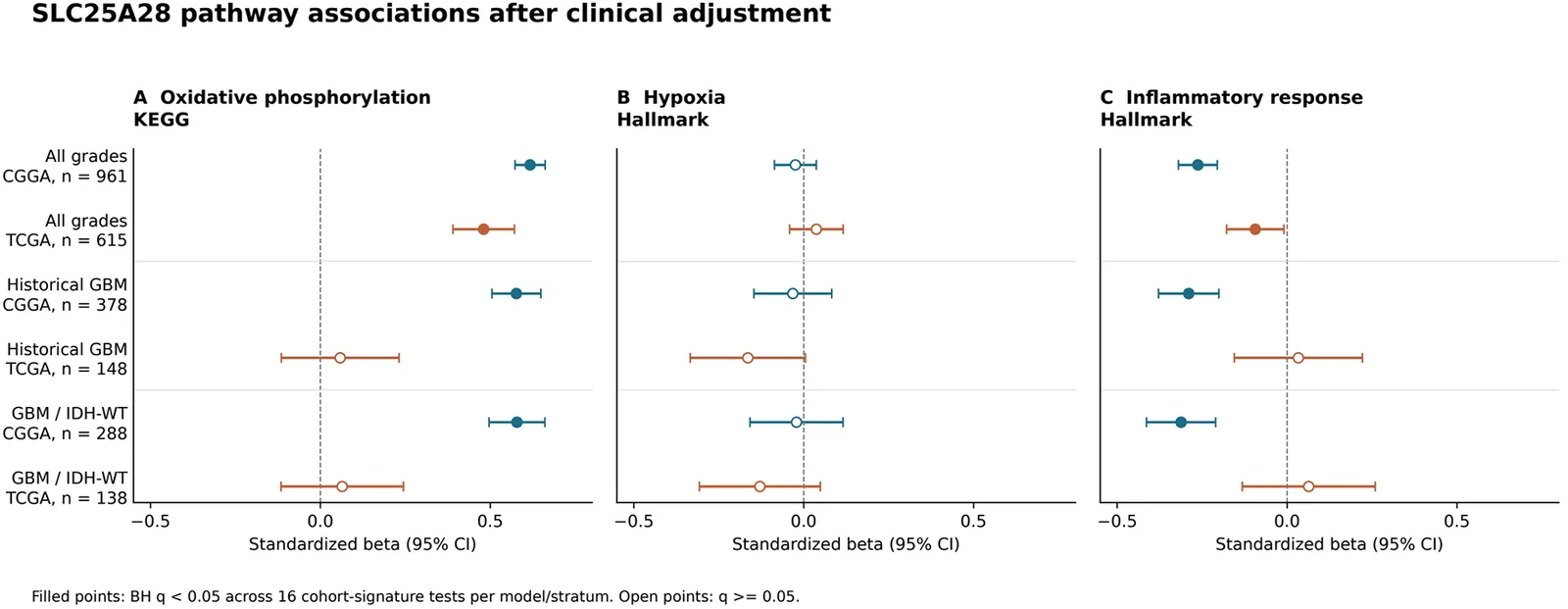
SLC25A28 pathway associations after clinical adjustment. Forest plots show standardized coefficients for continuous SLC25A28 expression in relation to **(A)** KEGG oxidative phosphorylation, **(B)** Hallmark hypoxia and **(C)** Hallmark inflammatory response. All-grade models adjust for categorical WHO grade and IDH status (CGGA n = 961; TCGA n = 615). Historical GBM models additionally account for age, sex and, in CGGA, primary/recurrent/secondary status, with IDH adjustment (n = 378 and 148). GBM/IDH-wild-type models include age, sex and, in CGGA, tumor status (n = 288 and 138). Predictor and outcome were standardized within each analytic population. Points represent coefficients and bars HC3 robust 95% confidence intervals; filled points denote BH q< 0.05 across eight signatures and two cohorts in the corresponding model/stratum. Open points denote q ≥ 0.05. Cohorts are shown separately. Historical GBM labels follow the source annotations. All eight signatures and additional strata are reported in **Supplementary Table S7**.

Hallmark hypoxia associations attenuated after grade/IDH adjustment on identical samples: coefficients changed from −0.114 to −0.025 in CGGA and from −0.333 to 0.037 in TCGA, with adjusted q values of 0.495 and 0.473. CGGA GBM showed evidence of nonlinearity (q = 1.10 × 10^-12^), and the apparent direction varied with the scoring approach and sample selection (**Supplementary Figure S7**). Immune-related pathway estimates also varied by cohort and signature. All eight signatures and sensitivity analyses are reported in **Supplementary Figure S8** and **Supplementary Tables S6**–**S8**.

### 3.6 SLC25A28 is associated with ferroptosis-related transcriptional features

To extend the metabolic characterization to iron-dependent cell-death pathways, we examined curated ferroptosis-related signatures. After grade/IDH adjustment, higher SLC25A28 expression was associated with a higher WikiPathways ferroptosis score in CGGA (standardized β = 0.066; 95% CI, 0.006–0.125; q = 0.036) and TCGA (β = 0.249; 0.153–0.345; q< 0.001). FerrDb driver GSVA scores were inversely associated with SLC25A28 in both cohorts (CGGA β = −0.357; −0.445 to −0.270; TCGA β = −0.445; −0.549 to −0.341; both q< 0.001). Suppressor-score associations were positive in CGGA (β = 0.238; 0.184–0.293; q< 0.001) and nonsignificant in TCGA (β = −0.053; −0.138–0.033; q = 0.228; **Figure 11**; **Supplementary Table S10**).

**Figure 11:**
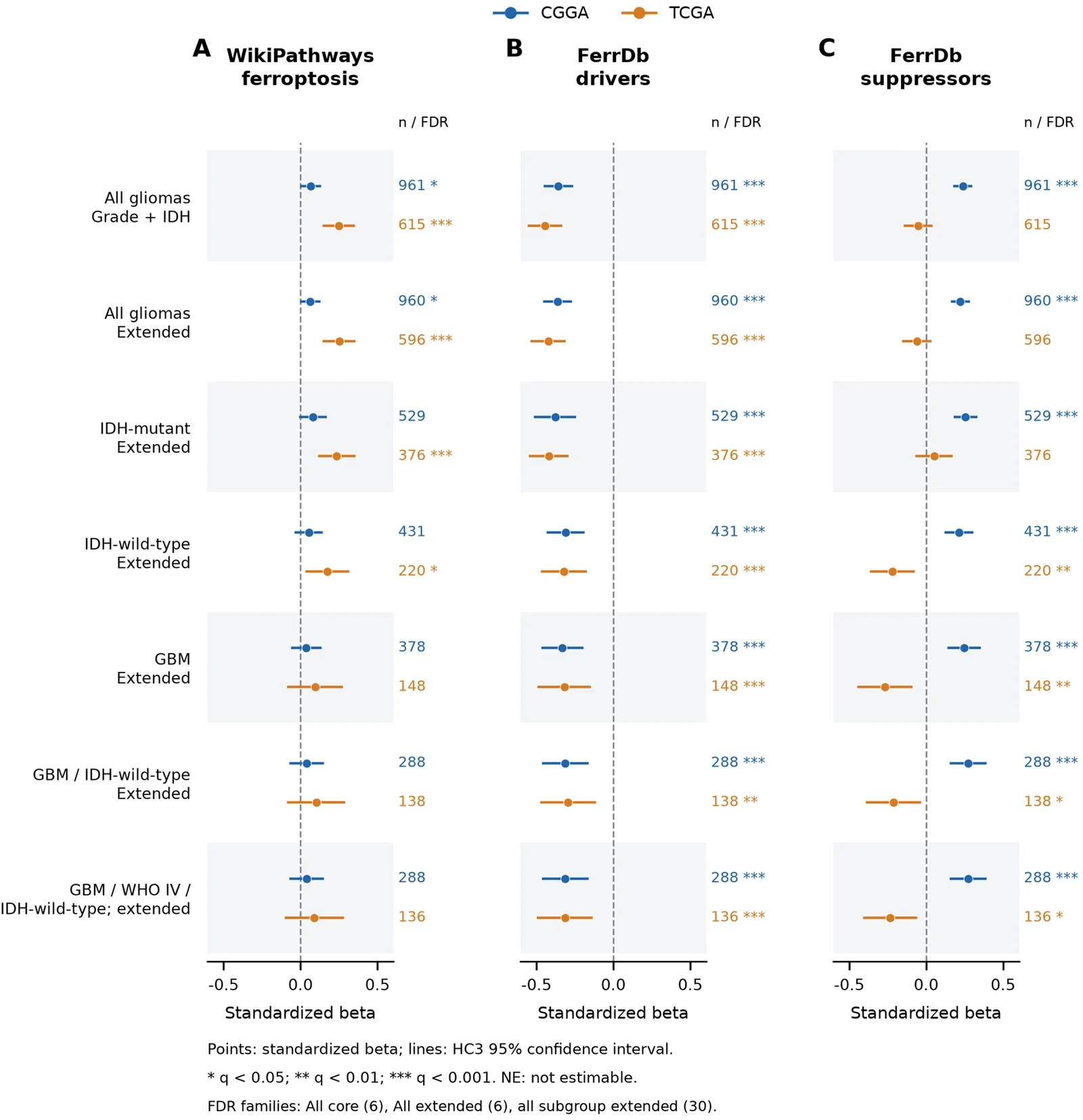
Adjusted associations between SLC25A28 expression and ferroptosis-related transcriptional signatures. Panels show associations with **(A)** the WikiPathways ferroptosis score (63 genes), **(B)** the FerrDb driver score (300 genes), and **(C)** the FerrDb suppressor score (549 genes). The same matched gene sets were used in both cohorts. Points indicate standardized regression coefficients, and horizontal lines indicate HC3-robust 95% confidence intervals. Vertical dashed lines indicate a coefficient of zero. CGGA and TCGA are shown in blue and orange, respectively. Core models adjust for WHO grade and IDH status. Extended models additionally adjust for age and sex and, in CGGA, recurrence status; covariates that are constant within a subgroup are omitted. Historical GBM is defined by the available histology annotation, and the strict subgroup requires GBM histology, WHO grade IV, and IDH-wild-type status. For each extended model in CGGA, the strict subgroup and the GBM/IDH-wild-type subgroup comprise the same 288 complete-case samples. SLC25A28 expression and signature scores are standardized within each complete-case analysis population. The displayed n indicates the number of samples analyzed for each estimate. Benjamini -Hochberg correction is applied separately to three testing families: six All/core tests, six All/extended tests, and 30 extended subgroup tests. Asterisks denote adjusted q values: *q < 0.05, **q < 0.01, and ***q < 0.001. NE, not estimable.

In extended GSVA models, inverse driver-score associations were also observed within historical GBM (CGGA β = −0.331; TCGA β = −0.318) and GBM/IDH-wild-type tumors (β = −0.312 and −0.293, respectively). In the latter population, CGGA included 288 patients (95% CI, −0.455 to −0.169; q = 6.23 × 10^-5^) and TCGA 138 patients (−0.466 to −0.120; q = 0.00194). The WikiPathways score did not show an FDR-significant linear association within GBM in either cohort, and suppressor scores showed opposite directions in the extended GBM models.

The positive all-glioma WikiPathways association in TCGA persisted across the specified sensitivity analyses, whereas the smaller CGGA association depended on scoring method and sample selection. For the driver set, the CGGA all-glioma coefficient changed from −0.361 with GSVA to 0.535 with mean-z scoring on the same 960 patients; direction reversals also occurred in GBM subsets. In TCGA, mean-z scoring retained the negative all-glioma driver association but did not support the inverse GSVA association within GBM subsets (**Supplementary Figure S9**). CGGA spline patterns and global-expression differences associated with 1p/19q-data availability further indicated sensitivity to expression distribution and case selection (**Supplementary Figure S10**; **Supplementary Table S11**).

Exploratory gene-level analysis identified 28 of 63 genes with FDR-significant associations in the same direction across both cohorts: 26 positive and two negative, including positive associations with GPX4, AIFM2, FTH1 and FTL. Seven additional genes were significant in both cohorts with opposite directions, including ACSL4 and NCOA4 (**Supplementary Figure S11**). These results associate SLC25A28 with ferroptosis-related transcriptional features while indicating dependence on signature definition, scoring method and cohort.

### 3.7 Immune-cell and checkpoint associations remain after multiplicity correction

After cohort-specific BH adjustment, differences in inferred immune-cell fractions remained significant for 10 of 22 populations in CGGA and 16 of 22 in TCGA (**Figure 12**; **Supplementary Table S5**). These results associate SLC25A28 expression with differences in the estimated immune composition of gliomas.

**Figure 12:**
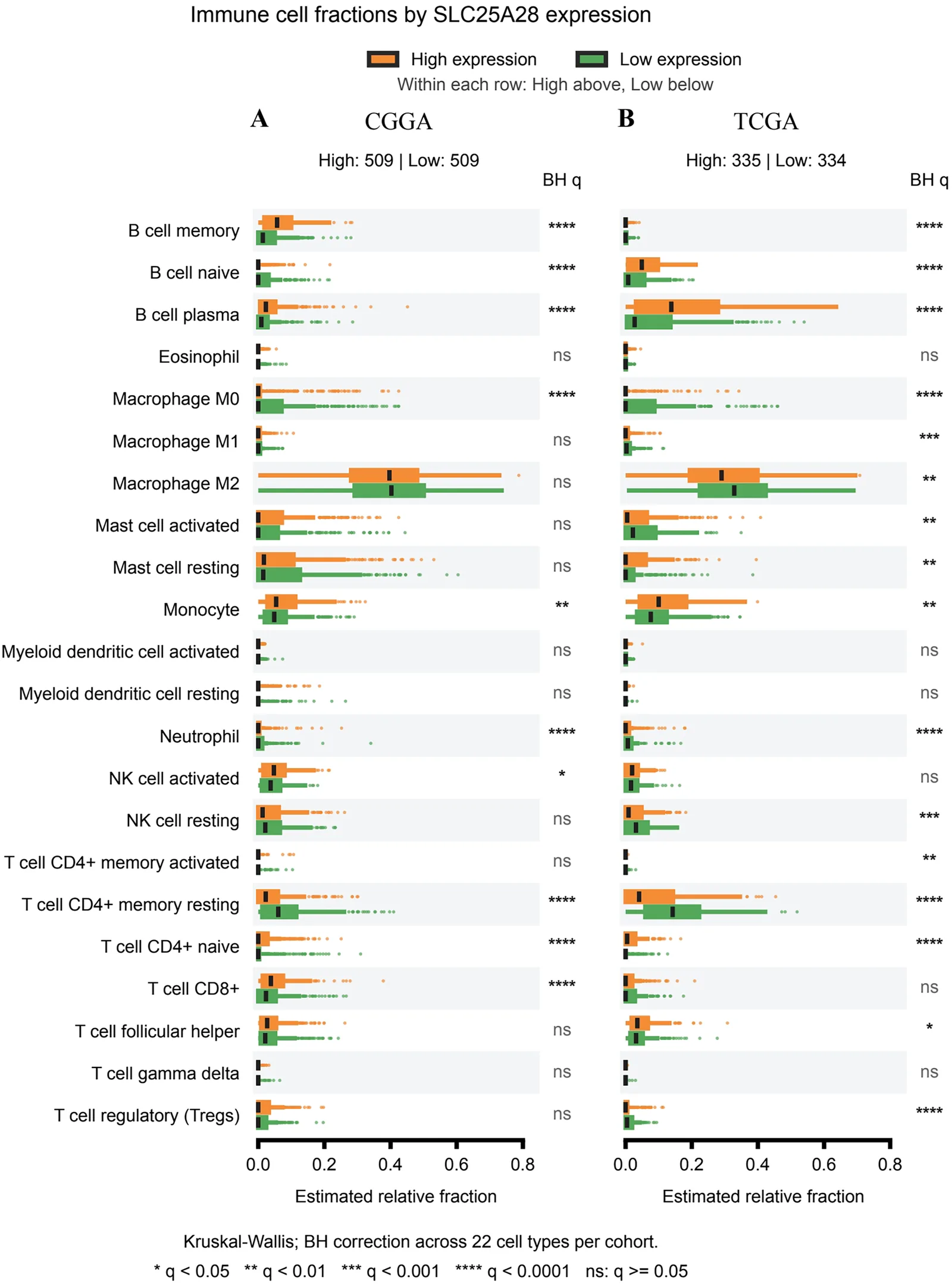
CIBERSORT-estimated immune-cell fractions by SLC25A28 expression. Panels show **(A)** the CGGA cohort, comprising 509 high-expression and 509 low-expression samples, and **(B)** the TCGA cohort, comprising 335 high-expression and 334 low-expression samples. Orange and green indicate the high- and low-expression groups, respectively; within each row, the high-expression group is displayed above the low-expression group. Boxes represent the interquartile range, black lines indicate the median, whiskers extend to the most extreme observations within 1.5 times the interquartile range, and points indicate outliers. Group differences are assessed using Kruskal -Wallis tests with tie correction. P values are adjusted using the Benjamini -Hochberg method across 22 immune-cell types separately within each cohort. Symbols denote adjusted q values: *q < 0.05, **q < 0.01, ***q < 0.001, and ****q < 0.0001; ns, q ≥ 0.05.

Four checkpoint correlations remained significant in CGGA (CTLA4, LAG3, VTCN1 and C10orf54), compared with all ten in TCGA (**Figure 13**). LAG3, VTCN1 and C10orf54 showed positive correlations in both cohorts, with CGGA/TCGA r values of 0.475/0.100, 0.099/0.121 and 0.395/0.210, respectively. CTLA4 showed weak associations of opposite direction (0.109/−0.117). The CGGA HAVCR2 association did not remain significant after adjustment (nominal P = 0.0443; q = 0.0839), and several negative correlations in TCGA were not supported in CGGA. The shared positive associations therefore coexist with broader cohort-specific checkpoint patterns.

**Figure 13:**
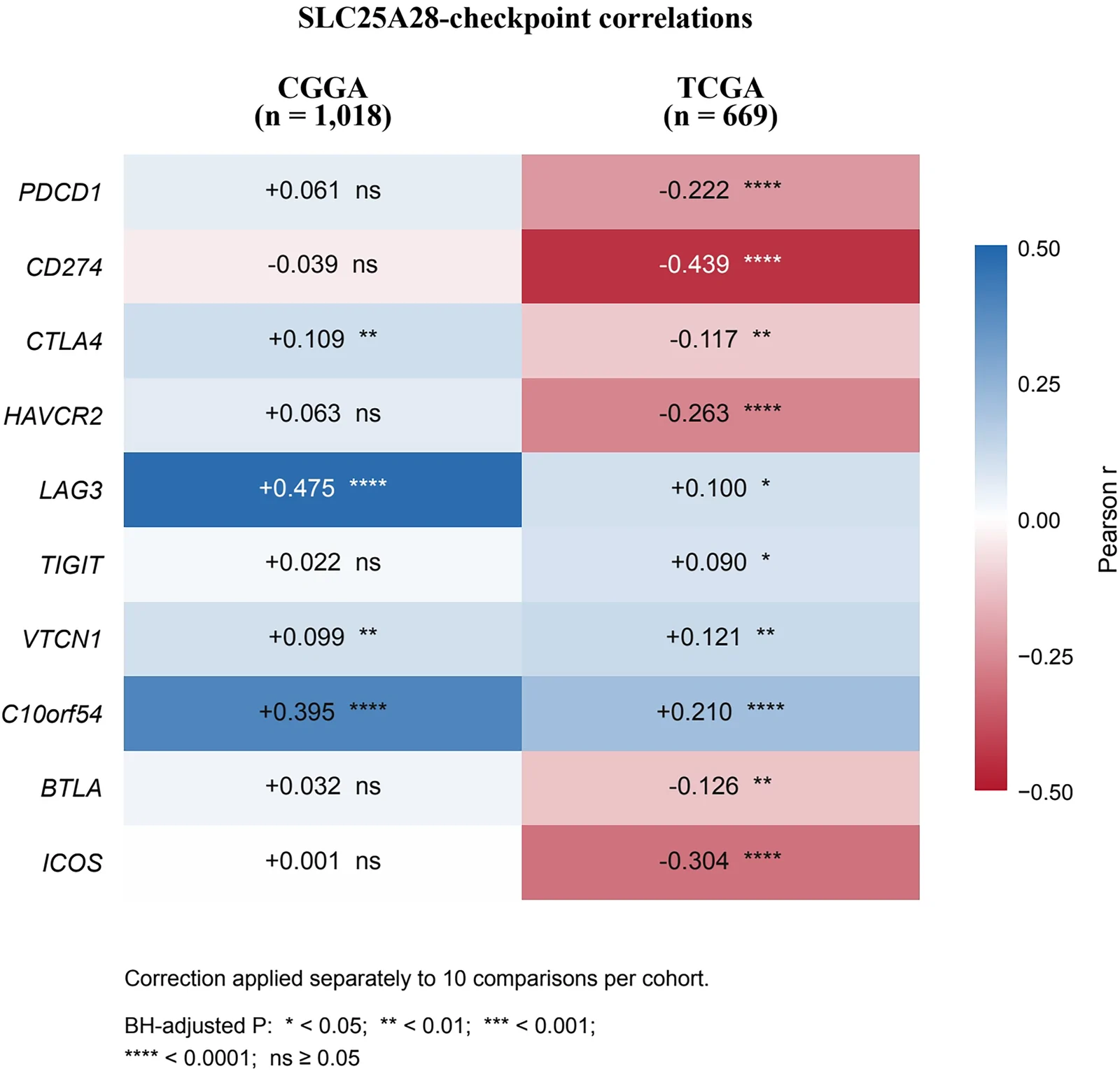
SLC25A28 expression and immune-checkpoint genes in CGGA (n=1,018) and TCGA (n=669). Tiles show Pearson correlation coefficients; blue denotes positive and red negative correlations on a common scale. Two-sided P values were BH-adjusted across ten checkpoint comparisons separately within each cohort. Symbols denote adjusted P values: *P<0.05, **P<0.01, ***P<0.001, ****P<0.0001; ns, P≥0.05. These are expression correlations without clinical-covariate or tumor-purity adjustment.

### 3.8 Experimental validation of SLC25A28 expression

qRT-PCR showed lower SLC25A28 mRNA expression in U87 and U251 glioma cells than in NHA cells (**Figure 14**).

**Figure 14:**
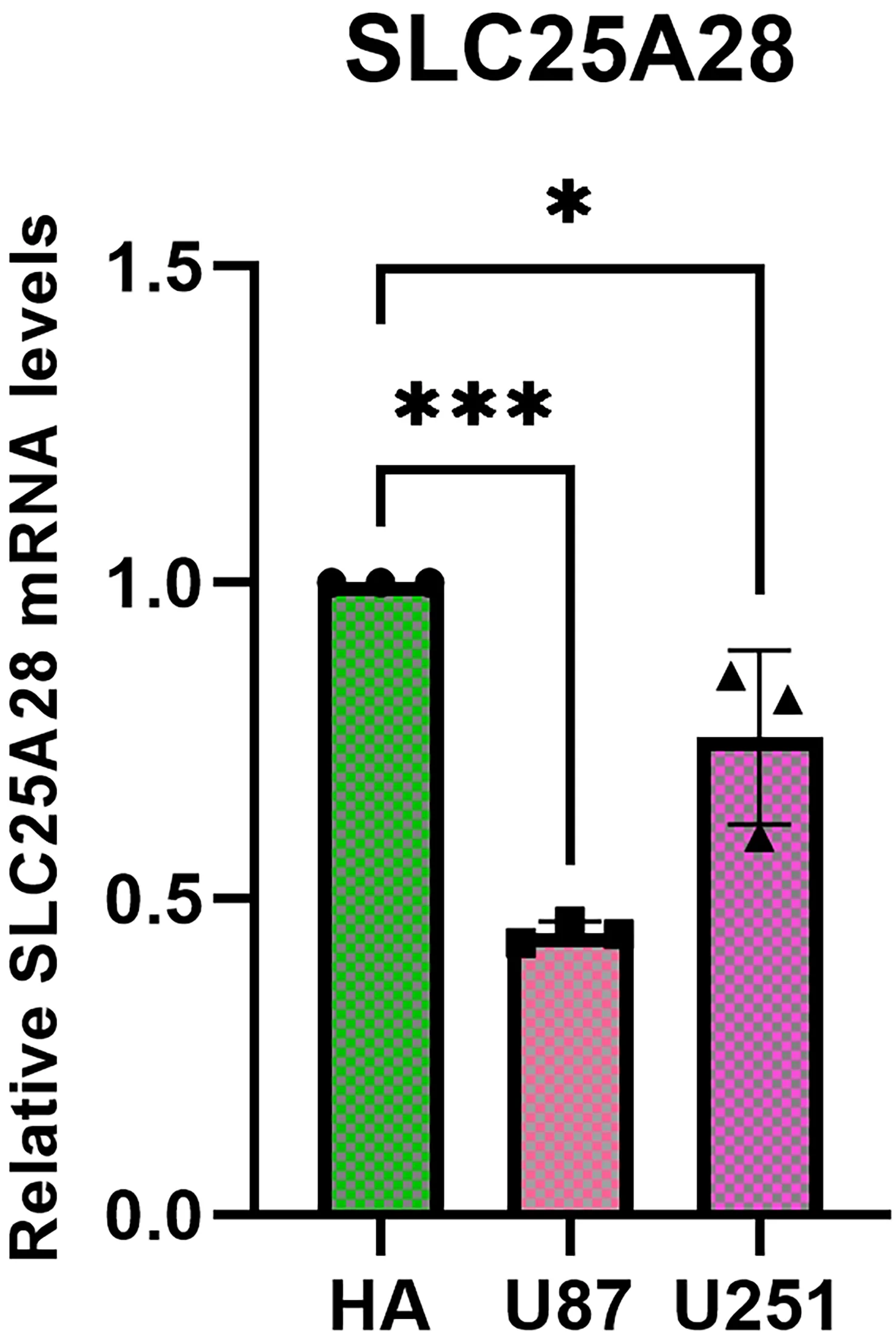
SLC25A28 mRNA expression in normal human astrocytes and glioma cell lines. Relative SLC25A28 mRNA levels were assessed by qRT-PCR in normal human astrocytes (HA in the plot), U87 cells, and U251 cells. Brackets indicate the groups being compared. Significance notation: *P < 0.05, **P < 0.01, and ***P < 0.001.

Western blotting likewise showed lower SLC25A28 protein abundance in U87 and U251 cells than in NHA cells (**Figure 15**). Together, the transcript and protein measurements consistently showed lower SLC25A28 expression in the two glioma cell lines.

**Figure 15:**
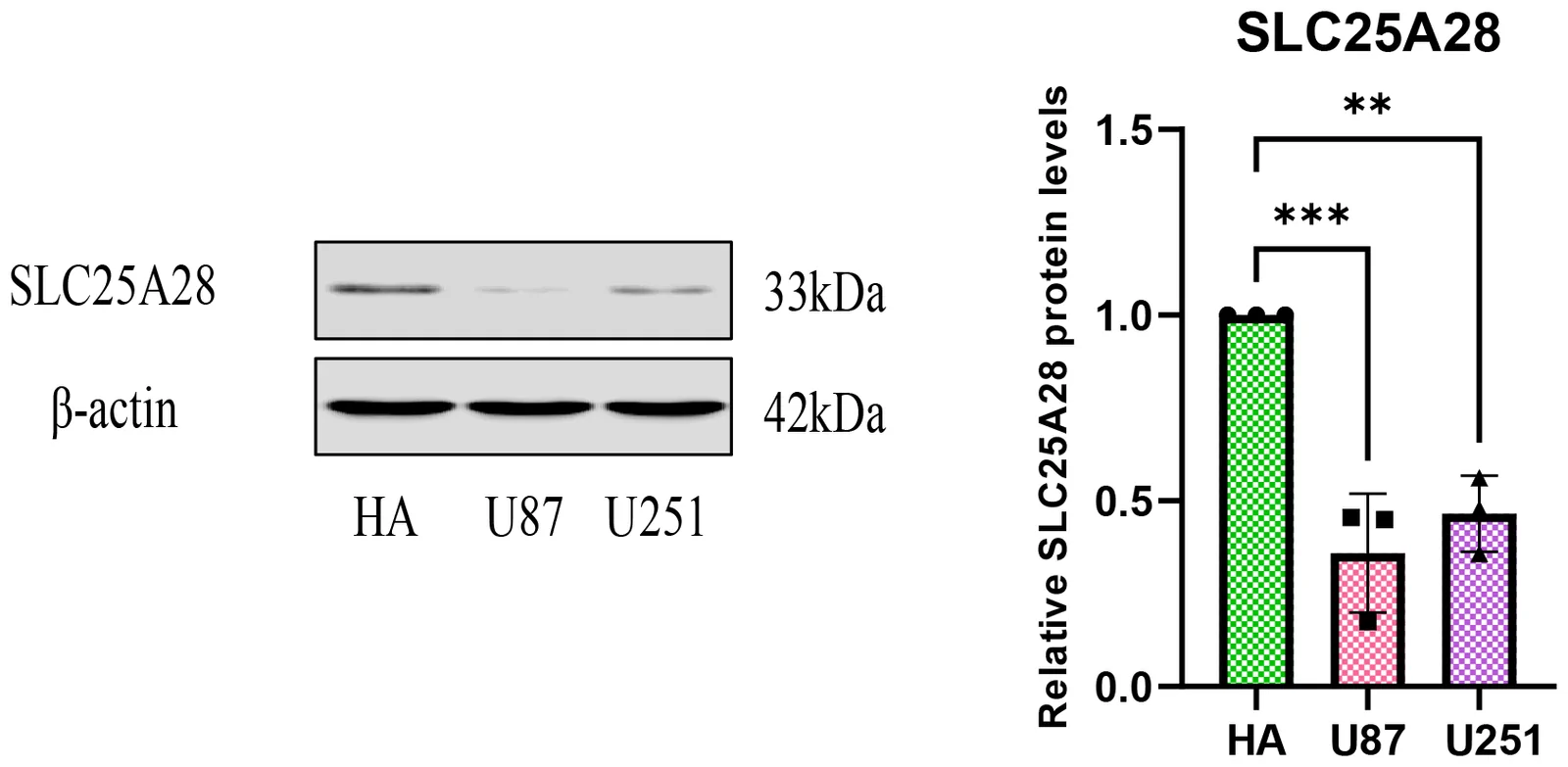
SLC25A28 protein expression in normal human astrocytes and glioma cell lines. The left panel shows SLC25A28 and β-actin immunoblots for normal human astrocytes (HA), U87 cells, and U251 cells. The right panel shows the corresponding relative SLC25A28 protein levels. β-actin serves as the loading control. Brackets in the quantification panel indicate the groups being compared. Significance notation: *P < 0.05, **P < 0.01, and ***P < 0.001.

## 4 Discussion

This study identifies SLC25A28 as a candidate prognostic biomarker associated with glioma clinicopathological characteristics, mitochondrial metabolism, ferroptosis-related transcriptional features and immune features. Lower expression was associated with adverse clinical and molecular characteristics and shorter overall survival across multiple cohorts. The persistence of oxidative-phosphorylation associations after grade/IDH adjustment in both pooled cohorts provides evidence of a metabolic relationship beyond the observed WHO-grade and IDH composition. Ferroptosis-focused analyses further identify a transcriptional association whose direction and strength depend on the signature and analytical setting. By integrating systematic SLC25-family screening, multi-cohort survival evidence, pathway and immune analyses, and expression validation, our findings extend previous work on mitochondrial iron transport and provide a clinical and transcriptomic basis for investigating SLC25A28 in glioma.

Mitochondrial transport provides a biologically relevant context for these findings. Mitochondria participate in metabolic homeostasis, cell survival and immune-cell function (5, 6, 28–32). SLC25A28 encodes mitoferrin-2, while SLC25A37 encodes mitoferrin-1; both contribute to iron delivery for heme and iron–sulfur-cluster synthesis, but their regulation and turnover differ (13–17). Prior glioma-cell work implicated mitoferrin-2 in arsenic trioxide-induced oxidative injury (18). Together with our oxidative-phosphorylation findings, this provides a rationale for studying SLC25A28 in mitochondrial energy metabolism and iron-dependent redox biology, while recognizing the treatment-specific context of the earlier experimental evidence.

The family-wide screen identified SLC25A1 and SLC25A28 in both cohorts, providing a statistical basis for investigating mitochondrial transport in glioma. SLC25A28 was prioritized for its direct relevance to iron import, while the mitochondrial citrate carrier SLC25A1 (33) and glutathione transporter SLC25A39 (34) represent complementary metabolic candidates. The distinct prognostic directions of SLC25A28 and SLC25A37 also suggest that shared mitochondrial iron transport activity need not imply identical associations with disease. Experimental work implicating mitoferrin-1 in glioblastoma proliferation (19) supports further comparison of their regulation and cellular contexts rather than assuming equivalent biological effects.

Lower SLC25A28 expression accompanied higher grade, IDH-wild-type status and 1p/19q non-codeletion, linking its expression to established clinical and molecular features (35–37). Clinical Cox analyses supported an association with survival after adjustment for the included covariates. The IDH-mutant and IDH-wild-type subgroup findings in both cohorts provide further exploratory support, while differences within GBM indicate that the strength of the association depends on disease setting. These observations favor evaluating SLC25A28 within molecularly characterized populations rather than treating diffuse glioma as a uniform disease.

The nine-cohort meta-analysis strengthens the prognostic evidence: low expression was associated with poorer survival, and no single cohort drove the pooled result. The weaker estimate in GBM-only datasets, together with the within-cohort subgroup findings, suggests that tumor composition can contribute to heterogeneity. The composition analysis was limited by the small number of GBM-only cohorts, and residual variation could also reflect platform, treatment, follow-up and molecular differences. The optimal-cutpoint analysis preserved the overall direction while illustrating the sensitivity of effect size and heterogeneity to the grouping strategy.

A principal biological finding is the positive association between SLC25A28 and oxidative-phosphorylation programs after adjustment for WHO grade and IDH status in both pooled cohorts. This result is consistent with the role of mitochondrial iron in heme and iron–sulfur-cluster synthesis and provides evidence of a metabolic relationship beyond these measured clinical characteristics. Persistence within CGGA GBM and GBM/IDH-wild-type tumors motivates mechanistic investigation in those settings, although the lack of a comparable association in TCGA GBM limits generalization. In contrast, the attenuation of hypoxia associations after clinical adjustment indicates a stronger contribution from tumor composition to that relationship.

The ferroptosis-focused analyses extend this metabolic context to curated pathway components and regulators. Ferroptosis involves iron availability, lipid peroxidation and antioxidant defenses (20, 21), processes relevant to the mitochondrial iron-transport function of SLC25A28. The consistent positive WikiPathways association across all-glioma sensitivity analyses in TCGA, together with clinically adjusted driver GSVA associations in both cohorts and their GBM subsets, supports further investigation of this transcriptional relationship. The pathway and regulator sets represent different collections of genes, however, and the CGGA scoring-method reversal, lack of mean-z support for the TCGA GBM driver association, and cohort-dependent suppressor patterns preclude a uniform pro-ferroptotic or anti-ferroptotic interpretation. Gene-level associations with GPX4, AIFM2 and iron-handling components provide additional description of this relationship within the same datasets. Demonstrating an effect on ferroptotic susceptibility requires gene perturbation and direct measurements of iron handling, lipid peroxidation and cell death.

The immune analyses identify associations that persist after correction for multiple testing. IL-18 has been shown to promote temozolomide resistance through PI3K/AKT activation and to increase CD274 (PD-L1) expression in glioma cells, linking inflammatory signaling with treatment resistance and immune-checkpoint expression (38). The positive LAG3, VTCN1 and C10orf54 correlations in both cohorts support a shared subset of immune-related features, while other checkpoint relationships vary across cohorts. Differences in grade/IDH composition, recurrent-tumor representation and cellular composition may contribute to this variation. Bulk-expression correlations cannot distinguish tumor-cell regulation from differences in immune or stromal abundance, so these findings provide a basis for focused cellular studies rather than evidence of direct checkpoint regulation or immunotherapy response.

The separation of survival by the SLC25A28-containing risk score, including in the IDH-mutant/WHO grade II–III subgroup, together with the time-dependent AUCs of the four-variable score in both cohorts, supports its potential for prognostic stratification and discrimination. TCGA provides a useful cross-cohort assessment, although its contribution to candidate selection means that a cohort untouched by the development process is still needed for independent validation. Non-proportional effects of grade and IDH constrain interpretation of the broader clinical models; diagnostics of the expression-and-age score apply specifically to that specification. Apparent calibration and individual-predictor decision curves provide descriptive performance information, and incremental clinical utility requires comparison with established clinical models.

This study is limited by its retrospective design, incomplete clinical annotations and complete-case selection. Historical diagnoses and outcome-selected subgroup cutpoints constrain the interpretation and generalizability of some subgroup findings. Residual confounding, including tumor purity and unmeasured treatment differences, may affect metabolic and immune associations. Ferroptosis-related estimates also depended on signature definition, scoring method and analysis population, particularly in CGGA; transcriptional scores cannot be equated with ferroptotic activity. Experimental validation confirms expression differences in U87 and U251 cells relative to normal human astrocytes, but patient-tissue studies and functional experiments are needed to establish clinical applicability and biological mechanisms.

## 5 Conclusions

SLC25A28 is a candidate prognostic biomarker associated with glioma clinicopathological characteristics, mitochondrial metabolism, ferroptosis-related transcriptional features and immune features. Multi-cohort survival evidence and grade/IDH-adjusted metabolic associations support its prognostic and biological relevance, while the ferroptosis findings identify a context-dependent transcriptional relationship for further investigation. These results extend research on mitochondrial iron transport in glioma and provide a foundation for evaluating SLC25A28 in molecularly defined patient populations. Independent clinical validation and functional studies will help establish its value for prognostic stratification and clarify the mechanisms underlying these associations.

## Data Availability

The raw data supporting the conclusions of this article will be made available by the authors, without undue reservation.
