# Supplementary material for: Prognostic significance of SLC25A28 and its associations with mitochondrial metabolism and immune features in glioma
Source: Front Oncol. 2026 Oct 30;16:1917001. doi: 10.3389/fonc.2026.1917001 (PMC13759410; doi:10.3389/fonc.2026.1917001)
Supplement: Supplementary file 1 [file DataSheet1.pdf]

Supplementary Material

Analytical details, cohort accounting, complete statistical results and additional figures supporting the study.

Supplementary Methods

Cohort accounting and survival-analysis populations

Supplementary Table S1 summarizes the 1,018 CGGA and 669 TCGA source records. Categorical percentages use the full source-cohort denominators and include missing categories; age is summarized as the median and first–third quartiles. Histology and WHO grade are distinct recorded variables. Source diagnoses and molecular annotations were used for subgroup assignment. Supplementary Table S2 reports variable completeness, sequential exclusions, subgroup sizes and cutoff definitions. Eligibility counts for specified variable combinations are distinguished from the sample sizes displayed in individual analyses.

Expression, overall-survival time and event status were recorded for 983 CGGA and 667 TCGA records. Requiring age, WHO grade and IDH status yielded 929 and 596 complete records, respectively. The full-cohort risk-score Kaplan–Meier displays in Figure 8 include 929 CGGA patients and 595 TCGA patients. Among the 596 TCGA records meeting the core completeness criteria, 595 had nonnegative follow-up and 593 had strictly positive follow-up. Across the complete TCGA survival records, one record had a negative recorded time and three censored records had zero recorded time; the core clinical subset included the negative-time record and two of the zero-time records. Supplementary Table S2 distinguishes recorded-survival completeness from nonnegative- and positive-time eligibility. CGGA had no nonpositive follow-up times among its records with complete survival information.

Cohort-level expression Kaplan–Meier analyses used each cohort's median SLC25A28 expression. The ten expression-based subgroup analyses in Figure 4 used the GlioVis optimal-cutpoint function within the relevant subgroup. These outcome-selected analyses were exploratory, and their nominal P values were not adjusted for cutpoint selection. Risk-score analyses instead used the median score within each displayed population. The six IDH-by-grade subgroup plots in Supplementary Figure S3 and the 415-patient IDH-mutant/WHO grade II–III plot in Figure 8 applied the CGGA-derived SLC25A28-plus-age score without estimating subgroup-specific coefficients. The latter plot contains 211 low-risk and 204 high-risk patients. All expression and risk-score cutoffs are identified separately in Supplementary Table S2.

Meta-analysis and cohort-composition sensitivity analyses

The primary random-effects meta-analysis combined the nine cohort-specific median-expression comparisons. Hazard ratios compare low with high SLC25A28 expression. Log hazard ratios and their standard errors were obtained from the cohort estimates and confidence intervals. Leave-one-out analyses recalculated the pooled association and heterogeneity after excluding each cohort in turn (Supplementary Figure S1). Input estimates and analysis versions are provided in Supplementary Table S4.

For the exploratory cohort-composition analysis in Figure 5, LeeY and Murat were classified as GBM-only datasets and the other seven datasets as mixed glioma datasets according to the GlioVis dataset descriptions. This was a post hoc dataset-level classification. The analysis used the median-split effect estimates at their reported precision. Random-effects subgroup estimates used restricted maximum likelihood (REML), with conventional Wald 95% confidence intervals for the forest-plot diamonds. A mixed-effects meta-regression included a GBM-only indicator, a common residual between-study variance and modified Hartung–Knapp inference with seven residual degrees of freedom; the variance multiplier was constrained to be at least one. The GBM-only subgroup contained two studies. Repository-level composition was not used as a substitute for individual patient-level adjustment.

A separate sensitivity analysis used cohort-specific GlioVis optimal expression cutoffs across the same nine datasets, followed by random-effects pooling and leave-one-out analysis (Supplementary Figure S2). Because these cutoffs were selected using survival outcomes, these

estimates were interpreted as exploratory. The median-cutoff and optimal-cutoff results were reported separately, including their respective heterogeneity and influence estimates.

Pathway definition and scoring

Pathway analyses used the GlioVis expression and phenotype exports dated 8 December 2023, comprising 1,018 matched CGGA records and 669 matched TCGA-GBMLGG records. The transformed expression values were used without further logarithmic transformation. Survival information was not required for these analyses. Eight signatures from MSigDB human release 2023.1 were examined: KEGG oxidative phosphorylation; GO hypoxia-inducible factor 1alpha signaling and adaptive immune response; and Hallmark oxidative phosphorylation, hypoxia, inflammatory response, interferon alpha response and interferon gamma response. Exact set identifiers and matched-gene coverage are provided in Supplementary Table S6.

Gene set variation analysis (GSVA) scores were calculated separately within each full cohort using GSEApy 1.3.1, with a Gaussian kernel, weight 1, mx_diff = False, abs_rnk = False and a permitted matched set size of 5–2,000 genes. These scores were held fixed for subsequent subgroup analyses. Constant genes were removed. Primary analyses used exact gene-symbol matching, and SLC25A28 was excluded from all signatures to avoid direct self-inclusion. The GO HIF1alpha signaling set contained five matched genes and was considered separately from the broader Hallmark hypoxia signature.

Scoring sensitivity analyses used the mean of gene-wise standardized expression values and GSVA after unambiguous HGNC previous-symbol mapping. Duplicate mapped features were averaged. Harmonized comparisons restricted both gene-set membership and the expression background to shared variable genes across the two cohorts.

Clinically adjusted pathway associations and robustness analyses

Ordinary least-squares models related pathway scores to continuous SLC25A28 expression. Predictor and outcome were standardized using the sample standard deviation within each model's analytic population. Primary models included categorical WHO grade and IDH status. Extended models additionally included age, sex and, for CGGA, primary/recurrent/secondary tumor status. Models restricted to historical GBM included IDH status; IDH-restricted models included grade when grade varied. Covariates constant within a subgroup were omitted. Analyses used complete cases without imputing clinical labels, and unadjusted and adjusted estimates were compared on identical samples. Subgroups with fewer than 30 complete cases were described but not modeled. The principal associations are presented in Figure 10, with complete estimates in Supplementary Table S7 and the eight-signature extended-model overview in Supplementary Figure S8.

Sensitivity analyses included grade-by-IDH cell fixed effects, individual grade/IDH strata, primary-tumor restriction, 1p/19q codeletion adjustment on matched samples, restriction to tumors simultaneously annotated as GBM, WHO grade IV and IDH-wild-type, rank-transformed regression, exclusion of the outer 1% of expression values, Huber regression and case-deletion influence analysis. Cohort-specific slope interactions assessed differences across grade and IDH groups. Differences between independently estimated cohort coefficients were also evaluated. Huber and case-deletion estimates were summarized descriptively.

Restricted cubic splines assessed nonlinear associations in GBM and GBM/IDH-wild-type subsets, with expression knots at the 5th, 35th, 65th and 95th percentiles. Nonlinear terms were tested jointly using HC3 robust Wald F tests. Alternative knots, expression-tail trimming, primary-tumor restriction, mean-standardized-expression scores and harmonized gene backgrounds were examined in hypoxia sensitivity analyses. Supplementary Figure S7 displays spline fits relative to median expression with pointwise HC3 95% confidence bands. Secondary expression-group comparisons used the 40th, 50th and 60th percentiles, defining the high group as expression strictly greater than the cutoff; these percentile cutoffs did not depend on survival outcomes.

Linear estimates were reported with HC3 heteroskedasticity-robust, t-based 95% confidence intervals and two-sided P values. Benjamini–Hochberg (BH) correction covered 16 cohort-signature tests within each named model/stratum. Separate BH families comprised 32 tests for spline nonlinearity, 32 tests for overall spline association, 96 percentile-cutoff tests, 32 slope-interaction tests, 24 between-cohort contrasts and 24 additional hypoxia nonlinearity sensitivity

tests. Harmonized scoring and matched-sample codeletion analyses used 16 tests per stratum or paired-codeletion model. Exploratory statistical evidence was defined as $q < 0.05$. Full sensitivity estimates are provided in Supplementary Table S8. Modeling used Python 3.12.14 and statsmodels 0.15.0.

Ferroptosis gene-set definition and scoring

Ferroptosis-focused analyses used the same CGGA and TCGA expression/phenotype exports as the metabolic pathway analyses, without another logarithmic transformation. The WikiPathways WP_FERROPTOSIS set contained 64 genes in MSigDB 2026.1.Hs. FerrDb V3 driver and suppressor records were restricted to the Ferroptosis category, human-organism annotations and protein-coding genes. All eligible confidence categories were retained for the primary FerrDb sets, including Validated, Screened, Deduced and Predicted annotations. Gene identities were checked against approved HGNC symbols, unambiguous previous symbols or identifier-supported exact aliases; unresolved identities were excluded. Ninety-two genes with both driver and suppressor roles were excluded from both directional sets, and SLC25A28 was excluded from all scores and their background. The resulting primary FerrDb lists contained 326 drivers and 565 suppressors before expression matching.

Expression features were harmonized using unambiguous HGNC previous-symbol mappings; duplicate mapped features were averaged. Scoring was restricted to 16,659 genes with nonzero variance present in both cohorts, with identical set membership across cohorts. Matched sets contained 63 WikiPathways genes, 300 drivers and 549 suppressors. GSVA used a Gaussian kernel, weight 1, mx_diff = True, abs_rnk = False and allowable matched set sizes of 5–3,000 genes. Scores were generated within each full cohort and held fixed across subgroups. The alternative mean-z score averaged gene-wise standardized expression calculated within that full cohort. The Validated-evidence sensitivity sets required at least one eligible human annotation rated Validated and were subsets of the primary exclusive sets; they retained 267 drivers and 510 suppressors after matching. No dual-role genes were restored. Definitions, matched membership, overlap and population accounting are provided in Supplementary Table S9. The three ferroptosis primary sets are distinct from the eight metabolic/immune signatures defined above and have their own multiplicity families.

Ferroptosis association models and robustness analyses

Primary models regressed each GSVA score on continuous SLC25A28, adjusting for categorical grade and IDH status; complete-case sample sizes were 961 in CGGA and 615 in TCGA. Extended models added age and sex and, for CGGA, primary/recurrent/secondary tumor status, yielding 960 and 596 samples. Both exposure and score were standardized using the sample standard deviation within each actual analytic population. Ordinary least-squares coefficients used HC3 robust covariance, t-based confidence intervals and two-sided P values. Unadjusted and core-adjusted comparators used the same samples as the corresponding adjusted models. Missing clinical variables were not imputed, and populations with fewer than 30 complete cases were not modeled.

Five overlapping subgroups were specified: IDH-mutant, IDH-wild-type, historical GBM, GBM/IDH-wild-type and strict GBM/WHO grade IV/IDH-wild-type. Core models adjusted for grade within IDH groups and for IDH within historical GBM; covariates fixed by subgroup definition were omitted. Extended subgroup models added age, sex and CGGA tumor status. Historical GBM extended models included 378 CGGA and 148 TCGA cases, and GBM/IDH-wild-type models included 288 and 138. The strict subgroup included the same 288 CGGA cases and 136 TCGA cases; the two CGGA definitions therefore do not provide independent replication. Model-specific population sizes, covariates and omissions are retained in Supplementary Tables S9 and S10.

Sensitivity models in all gliomas, historical GBM and GBM/IDH-wild-type used mean-z scoring, average-rank transformations of exposure and score, removal of values outside the complete-case 1st and 99th expression percentiles, restriction to primary CGGA tumors, additional 1p/19q adjustment, and the two Validated-evidence scores. For 1p/19q analysis, an extended reference model and a model adding 1p/19q were fitted to identical complete cases, so sample-selection effects could be distinguished from covariate adjustment. Restricted cubic splines in the same three populations used four knots at the 5th, 35th, 65th and 95th exposure percentiles. HC3 Wald

F tests evaluated the two nonlinear components and all three exposure components separately. Curves show score differences relative to the within-population median and are restricted to the 2.5th–97.5th exposure percentiles. Exploratory gene-level models related each of the 63 matched WikiPathways genes to SLC25A28 after grade/IDH adjustment; these describe the same expression data and do not constitute independent validation.

Separate BH families comprised six all-glioma primary tests, six all-glioma extended tests, 30 core subgroup tests, 30 extended subgroup tests, all 111 sensitivity tests, 18 spline nonlinearity tests, 18 overall spline tests, 36 between-cohort standardized-coefficient contrasts and all 126 gene–cohort tests. The sensitivity family included the paired 1p/19q reference estimates. Descriptive same-sample unadjusted/core comparisons did not receive separate q values. The planned denominator was retained if a model could not be estimated; no such exclusions occurred in the completed linear analyses. Full results are in Supplementary Tables S10–S11 and Figures S9–S11.

Descriptive assessment of CGGA expression distributions

Following sensitivity discordance, post hoc descriptive checks compared expression distributions by 1p/19q-data availability. Among all 1,018 CGGA source samples, the 78 without 1p/19q annotations had mean SLC25A28 expression of 2.373, compared with 5.679 in the 940 with annotations. The corresponding averages of per-sample mean expression across shared genes were 1.366 and 2.880. In the extended complete-case analysis, restriction from 960 cases to 886 with 1p/19q data changed the WikiPathways coefficient from 0.063 to −0.177 before adding 1p/19q to the model; the additional adjustment yielded −0.197. Thus, sample selection itself contributed to the observed change. The available phenotype file contained no verified series or batch variable. These descriptive checks did not change the expression values, main analysis population or model specification, and do not establish a technical or biological cause for the distributions (Supplementary Table S11).

Immune-cell comparisons and checkpoint correlations

Immune-cell comparisons used the expression-group labels in the CIBERSORT exports: 509 high-expression and 509 low-expression CGGA records, and 335 high-expression and 334 low-expression TCGA records. These are expression groups, not prognostic-risk groups. Kruskal–Wallis tests included tie correction. Two-sided Pearson tests assessed the associations of continuous SLC25A28 expression with the ten immune-checkpoint genes. BH correction included every test in the corresponding family, separately for the 22 immune-cell comparisons and ten checkpoint correlations in each cohort. Both nominal and adjusted P values are provided in Supplementary Table S5, with $q < 0.05$ as the significance threshold.

The CGGA cell-fraction comparisons used the grouping labels attached to the CIBERSORT export. The numerical threshold underlying those labels was not recoverable from the separate expression matrix used for checkpoint correlations. TCGA cell-fraction labels corresponded to its cohort expression median. The immune-cell and checkpoint analyses were unadjusted for clinical covariates and tumor purity.

Risk-score diagnostics, calibration and decision curves

The whole-cohort curves in Figure 8A/B used the CGGA-derived four-variable score described in the main Methods. The subgroup curves in Figure 8C and Supplementary Figure S3 instead used a separate CGGA score containing SLC25A28 expression and age, with fixed coefficients and subgroup-specific score medians. Schoenfeld-residual diagnostics in Supplementary Figure S4 assessed proportional hazards for this expression-and-age model. These diagnostics concern the two evaluated covariates; grade and IDH proportional-hazards tests belong to the separate clinical Cox analyses.

Calibration curves compared predicted and observed overall-survival probabilities at 1, 3 and 5 years in the CGGA development population (Supplementary Figure S5). Because evaluation used the development sample, these curves describe apparent calibration. Decision curves assessed SLC25A28, age, IDH status and WHO grade individually at 1, 3 and 5 years in CGGA and TCGA, using treat-all and treat-none reference strategies (Supplementary Figure S6). These single-predictor curves were separate from the combined-model score evaluation.

Supplementary tables

The full machine-readable tables are provided in the accompanying Supplementary_Tables directory, grouped by the table numbers below. Selected compact summaries are reproduced here; all remaining rows are retained in the CSV files.

Table S1 Cohort characteristics

Age is median (Q1–Q3). All other entries are counts and percentages of source records. Missing categories are included; absent treatment annotations are not coded as untreated.

Characteristic	Category	CGGA	TCGA
Age	Median (Q1–Q3), years	42 (35–51)	47 (35–59)
Records	Total	1,018	669
Sex	Male	601 (59.0%)	355 (53.1%)
Sex	Female	417 (41.0%)	254 (38.0%)
Sex	Missing	0 (0.0%)	60 (9.0%)
WHO grade	IV	388 (38.1%)	150 (22.4%)
WHO grade	III	334 (32.8%)	244 (36.5%)
WHO grade	II	291 (28.6%)	226 (33.8%)
WHO grade	Missing	5 (0.5%)	49 (7.3%)
Historical histology	GBM	388 (38.1%)	152 (22.7%)
Historical histology	Anaplastic Astrocytoma	214 (21.0%)	0 (0.0%)
Historical histology	Astrocytoma	175 (17.2%)	194 (29.0%)
Historical histology	Oligodendroglioma	112 (11.0%)	191 (28.6%)
Historical histology	Anaplastic Oligodendrolgioma	94 (9.2%)	0 (0.0%)
Historical histology	Anaplastic Oligoastrocytoma	21 (2.1%)	0 (0.0%)
Historical histology	Oligoastrocytoma	9 (0.9%)	130 (19.4%)
Historical histology	Missing	5 (0.5%)	2 (0.3%)
IDH status	Mutant	531 (52.2%)	429 (64.1%)
IDH status	Wild type	435 (42.7%)	233 (34.8%)
IDH status	Missing	52 (5.1%)	7 (1.0%)
1p/19q	Non-codeleted	728 (71.5%)	494 (73.8%)
1p/19q	Codeleted	212 (20.8%)	169 (25.3%)
1p/19q	Missing	78 (7.7%)	6 (0.9%)
MGMT promoter	methylated	472 (46.4%)	477 (71.3%)
MGMT promoter	unmethylated	376 (36.9%)	161 (24.1%)
MGMT promoter	Missing	170 (16.7%)	31 (4.6%)

Characteristic	Category	CGGA	TCGA
Disease stage	Primary	651 (63.9%)	616 (92.1%)
Disease stage	Recurrent	333 (32.7%)	0 (0.0%)
Disease stage	Secondary	30 (2.9%)	0 (0.0%)
Disease stage	Missing	4 (0.4%)	53 (7.9%)
Radiotherapy	Yes	767 (75.3%)	0 (0.0%)
Radiotherapy	No	164 (16.1%)	0 (0.0%)
Radiotherapy	Missing	87 (8.5%)	669 (100.0%)
Chemotherapy	Yes	635 (62.4%)	0 (0.0%)
Chemotherapy	No	275 (27.0%)	0 (0.0%)
Chemotherapy	Missing	108 (10.6%)	669 (100.0%)

Files: TableS1_cohort_characteristics.csv

Table S2 Sample accounting and cutoff definitions

Includes source completeness, missingness, subgroup sizes, plotted group reconciliation and cutoff registers. Model sizes are not inferred from variable-set reconstructions.

Population	CGGA	TCGA
Source records	1,018	669
Recorded survival	983	667
Core complete cases	929	596
Missing survival excluded	35	2
Additional core covariate exclusions	54	71

Files: TableS2_01_model_sample_counts.csv; TableS2_02_sequential_exclusions.csv; TableS2_03_variable_missingness.csv; TableS2_05_subgroup_sample_counts.csv; TableS2_06_KM_population_and_cutoff_reconciliation.csv; TableS2_08_cutoff_register.csv

Table S3 Comparison of four candidate genes

Univariable and SLC25-family multivariable Cox estimates. Family models contain 30 genes in CGGA and 31 in TCGA and do not adjust clinical covariates. Across-gene HR magnitude is not a predictive-performance ranking.

The compact summary below shows the SLC25-family multivariable estimates only; the full CSV also includes the univariable estimates.

Gene	CGGA HR (CI); P	TCGA HR (CI); P
SLC25A28	0.65 (0.53–0.80); <0.001	0.47 (0.29–0.76); 0.0021
SLC25A1	0.75 (0.64–0.88); <0.001	0.61 (0.45–0.83); 0.0018
SLC25A39	1.9 (1.5–2.4); <0.001	0.73 (0.43–1.2); 0.25
SLC25A37	1.1 (0.97–1.2); 0.14	1.7 (1.2–2.3); <0.001

Files: TableS3_candidate_gene_comparison.csv

Table S4 Meta-analysis inputs and sensitivity results

Primary median-split inputs, REML and DL subgroup results, meta-regression, and clearly separated optimal-cutoff summaries. Any dataset identifiers refer to source repositories; analysis populations are the GlioVis exports.

Version	HR (95% CI)	I^2
Original median	1.91 (1.46–2.50)	84.9%
Optimal cutoff	2.62 (2.06–3.34)	70.0%
Optimal, TCGA omitted	2.37 (2.04–2.76)	28.6%

Files: TableS4_meta_inputs.csv; TableS4_meta_pooled_results.csv; TableS4_meta_regression.csv; TableS4_meta_versions.csv; TableS4_optimal_cutoff_cohort_estimates.csv; TableS4_dataset_identifiers.csv

Table S5 Complete immune tests and BH correction

All 44 immune-cell tests and 20 checkpoint correlations, including nonsignificant results. BH families are separate by cohort and analysis type. None of these tests removes grade/IDH/purity confounding.

BH-significant tests	CGGA	TCGA
Immune cells	10/22	16/22
Checkpoints	4/10	10/10

Files: TableS5_immune_cell_BH_results.csv; TableS5_checkpoint_BH_results.csv

Table S6 Pathway populations and gene-set coverage

Input quality, clinical missingness, grade/IDH distribution and exact/harmonized gene coverage. The HIF1alpha set has only five matched genes and is distinct from broad Hallmark hypoxia.

Files: TableS6_input_quality_checks.csv; TableS6_clinical_missingness.csv; TableS6_grade_IDH_counts.csv; TableS6_gene_set_coverage.csv; TableS6_harmonized_gene_coverage.csv; TableS6_sample_flow.csv

Table S7 Complete continuous-expression pathway associations

All fitted targeted associations, including coefficient, HC3 interval, P, q, model, sample size and covariates. Predictor and outcome are standardized within each model population.

Files: TableS7_pathway_associations.csv

Table S8 Pathway sensitivity and heterogeneity analyses

Spline tests, cutoff sensitivity, harmonization, robust and influence analyses, paired codeletion adjustment, slope interactions and between-cohort contrasts. BH families are defined in the Methods; Huber and case-deletion estimates are descriptive.

Files: TableS8_between_cohort_differences.csv; TableS8_codeletion_paired_comparison.csv; TableS8_cutpoint_sensitivity.csv; TableS8_harmonized_sensitivity.csv; TableS8_hypoxia_nonlinearity_sensitivity.csv; TableS8_influence_robustness.csv; TableS8_nonlinearity.csv; TableS8_slope_interactions.csv

Table S9 Ferroptosis gene sets and analysis populations

The same matched genes were used in CGGA and TCGA. The five listed scores comprise three primary sets and two strict-evidence sensitivity sets. Full membership, source confidence annotations, shared background quality, clinical completeness and model-specific sample accounting are supplied in the accompanying files. SLC25A28 and genes with ambiguous identities were excluded; driver/suppressor sets exclude dual-role genes.

Gene set	Defined genes	Matched genes
WikiPathways ferroptosis	64	63
FerrDb drivers	326	300
FerrDb suppressors	565	549
FerrDb drivers, Validated	276	267
FerrDb suppressors, Validated	524	510

Files: TableS9_ferroptosis_gene_set_coverage.csv; TableS9_ferroptosis_scoring_gene_membership.csv; TableS9_ferroptosis_gene_set_overlap.csv; TableS9_ferroptosis_input_quality_checks.csv; TableS9_ferroptosis_clinical_missingness.csv; TableS9_ferroptosis_grade_IDH_counts.csv; TableS9_ferroptosis_sample_flow.csv; TableS9_ferroptosis_FerrDb_gene_evidence.csv

Table S10 Ferroptosis signature associations

Primary standardized coefficients after categorical WHO grade and IDH adjustment are summarized below. All six tests share one BH family. Complete primary, extended and subgroup estimates, including same-sample descriptive comparisons, are provided in the files. Positive coefficients denote higher signature scores with higher SLC25A28 expression, not increased ferroptosis activity.

Signature	Cohort	n	β (95% CI)	q
WikiPathways ferroptosis	CGGA	961	0.066 (0.006, 0.125)	0.0361
FerrDb drivers	CGGA	961	-0.357 (-0.445, -0.270)	7.15e-15
FerrDb suppressors	CGGA	961	0.238 (0.184, 0.293)	2.42e-16
WikiPathways ferroptosis	TCGA	615	0.249 (0.153, 0.345)	7.76e-07
FerrDb drivers	TCGA	615	-0.445 (-0.549, -0.341)	7.56e-16
FerrDb suppressors	TCGA	615	-0.053 (-0.138, 0.033)	0.228

Files: TableS10_ferroptosis_model_estimates.csv; TableS10_ferroptosis_primary_results.csv

Table S11 Ferroptosis robustness and gene-level results

All 111 sensitivity estimates, spline tests and curve coordinates, between-cohort coefficient contrasts, all 126 WikiPathways gene–cohort tests and descriptive post hoc CGGA expression checks are retained. BH families are defined in the Supplementary Methods. Nonsignificant and directionally discordant estimates are included; the post hoc expression checks are descriptive and do not introduce another hypothesis-testing family.

Files: TableS11_ferroptosis_nonlinearity.csv; TableS11_ferroptosis_spline_curves.csv; TableS11_ferroptosis_cohort_coefficient_differences.csv; TableS11_ferroptosis_WP_gene_level_associations.csv; TableS11_ferroptosis_posthoc_CGGA_expression_QC.csv; TableS11_ferroptosis_sensitivity_models.csv

Figure S1

Supplementary Figure S1. Leave-one-out sensitivity analysis of the meta-analysis

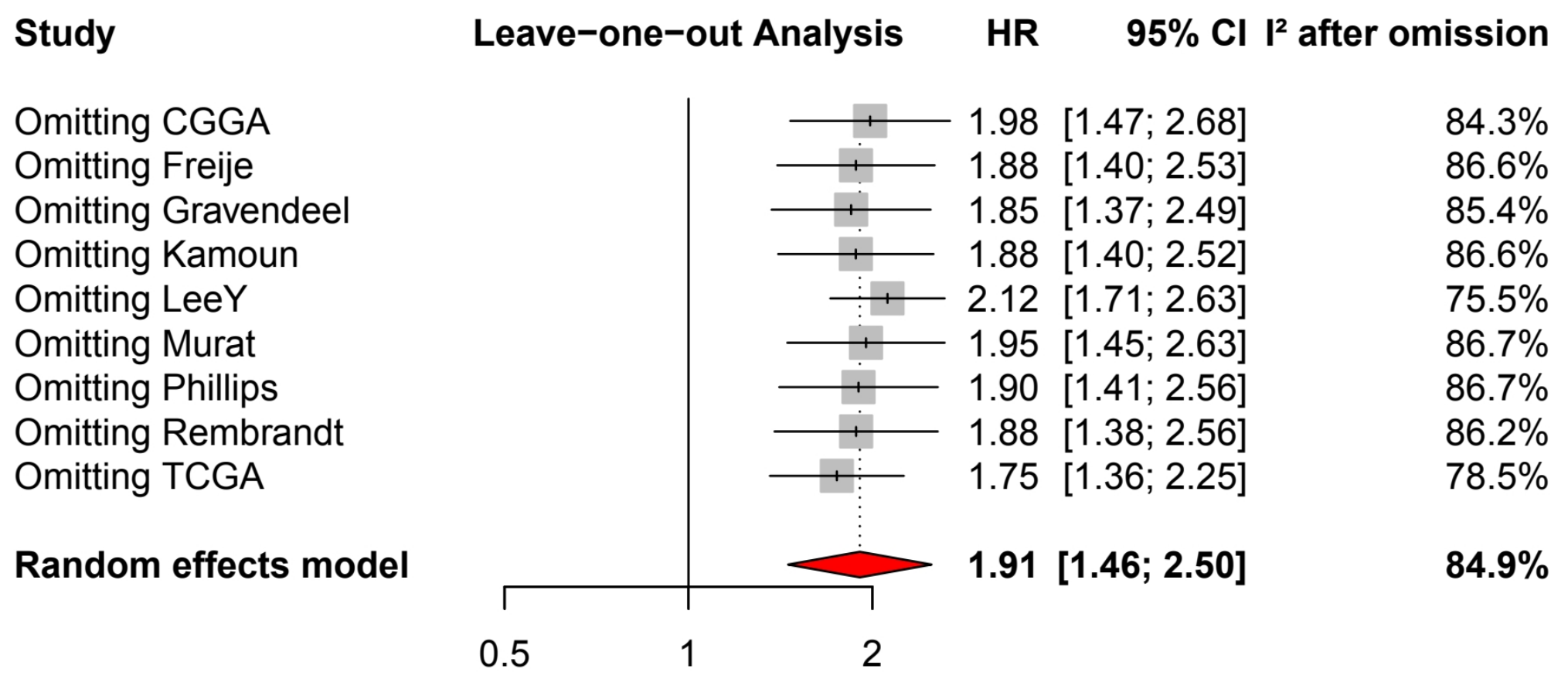

The pooled hazard ratio (HR) and 95% confidence interval (CI) were recalculated after sequential exclusion of each of the nine glioma cohorts. The overall random-effects estimate is shown by the red diamond. I-squared represents the proportion of variability attributable to between-cohort heterogeneity.

Figure S1. Leave-one-out sensitivity analysis of the primary nine-cohort random-effects meta-analysis using cohort-specific median SLC25A28 expression cutoffs. Each estimate excludes the indicated cohort. Hazard ratios compare low with high expression; horizontal bars indicate 95% confidence intervals. The pooled association remained positive, while substantial residual heterogeneity persisted.

Figure S2A

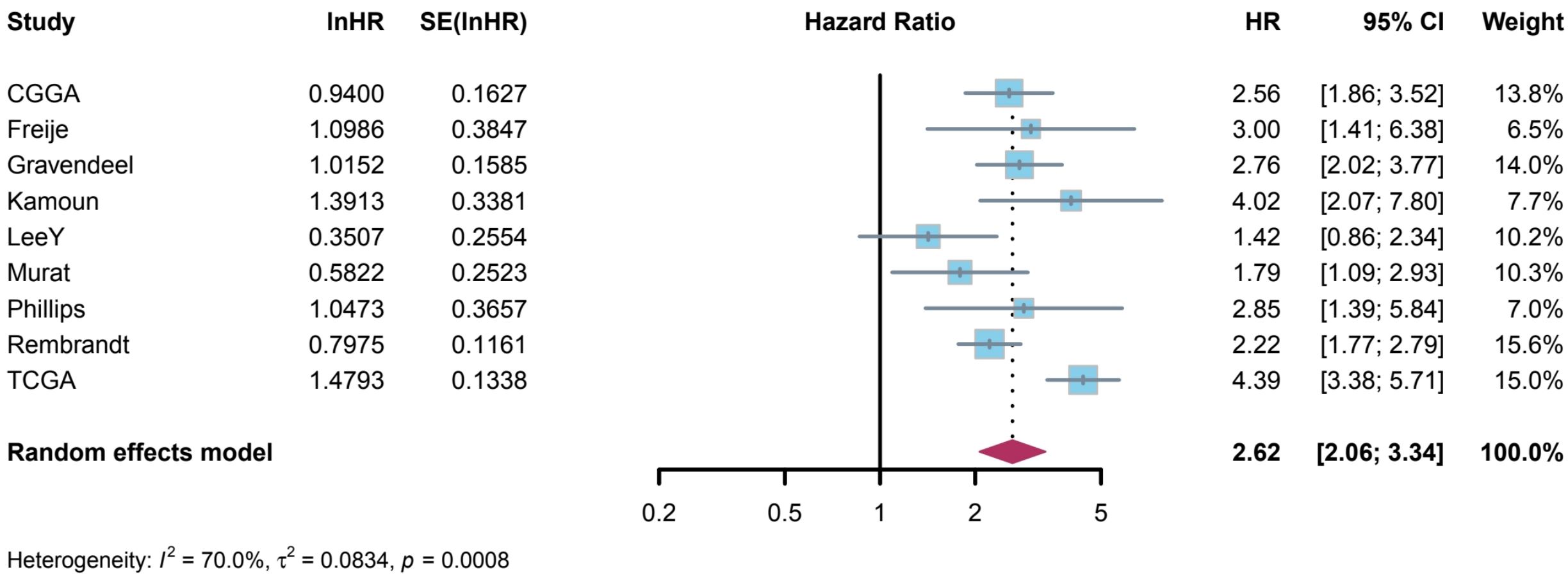

Figure S2A. Sensitivity analysis using cohort-specific optimal SLC25A28 expression cutoffs. (A) Random-effects meta-analysis across nine GlioVis cohorts. (B) Leave-one-out analysis of the same optimal-cutpoint estimates. Hazard ratios compare low with high expression; bars and diamonds show 95% confidence intervals. The pooled hazard ratio was 2.62 (95% CI 2.06–3.34), with I^2=70.0%. Excluding TCGA yielded HR 2.37 (95% CI 2.04–2.76) and I^2=28.6%. Optimal cutoffs were selected using survival outcomes, so effect estimates and nominal inference may be optimistic. These analyses supplement the median-cutoff main analysis.

Figure S2B

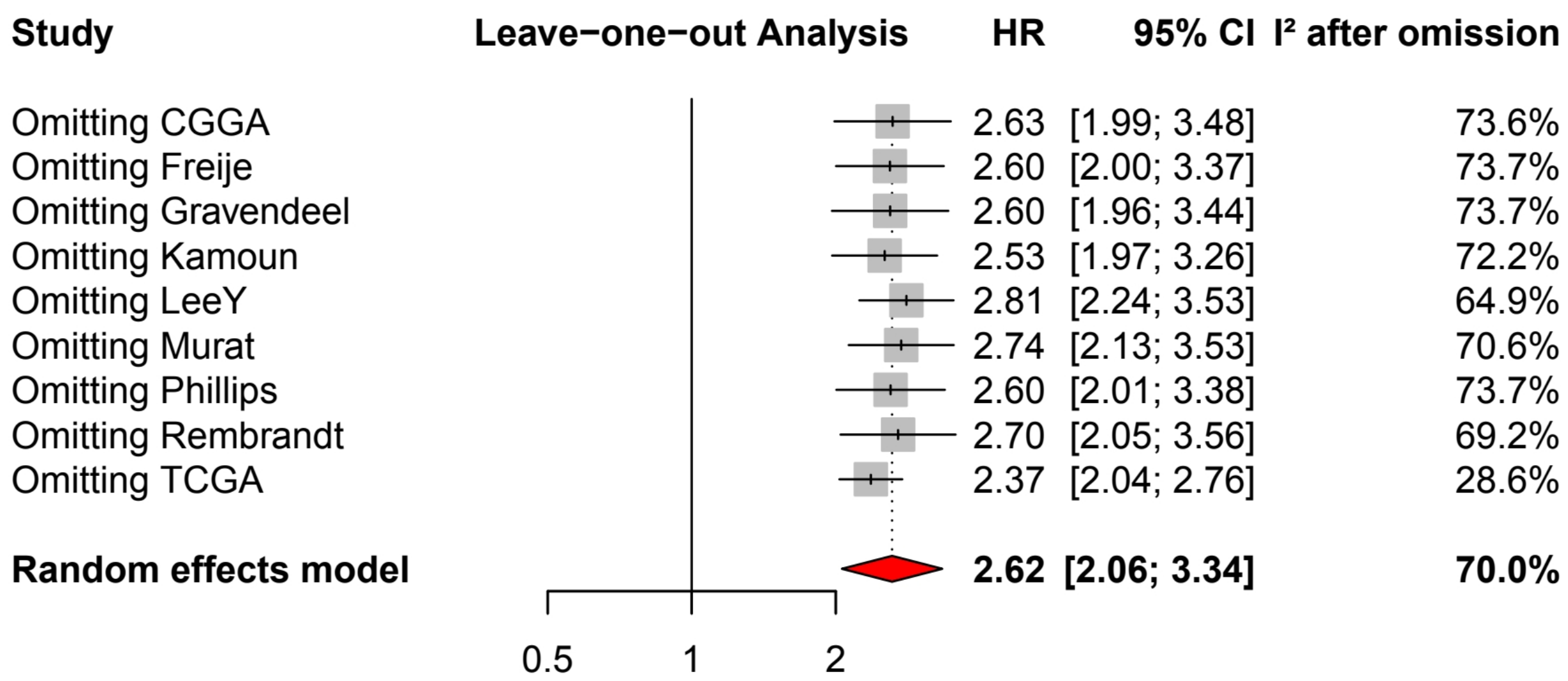

Figure S2B. Sensitivity analysis using cohort-specific optimal SLC25A28 expression cutoffs. (A) Random-effects meta-analysis across nine GlioVis cohorts. (B) Leave-one-out analysis of the same optimal-cutpoint estimates. Hazard ratios compare low with high expression; bars and diamonds show 95% confidence intervals. The pooled hazard ratio was 2.62 (95% CI 2.06–3.34), with I^2=70.0%. Excluding TCGA yielded HR 2.37 (95% CI 2.04–2.76) and I^2=28.6%. Optimal cutoffs were selected using survival outcomes, so effect estimates and nominal inference may be optimistic. These analyses supplement the median-cutoff main analysis.

Figure S3

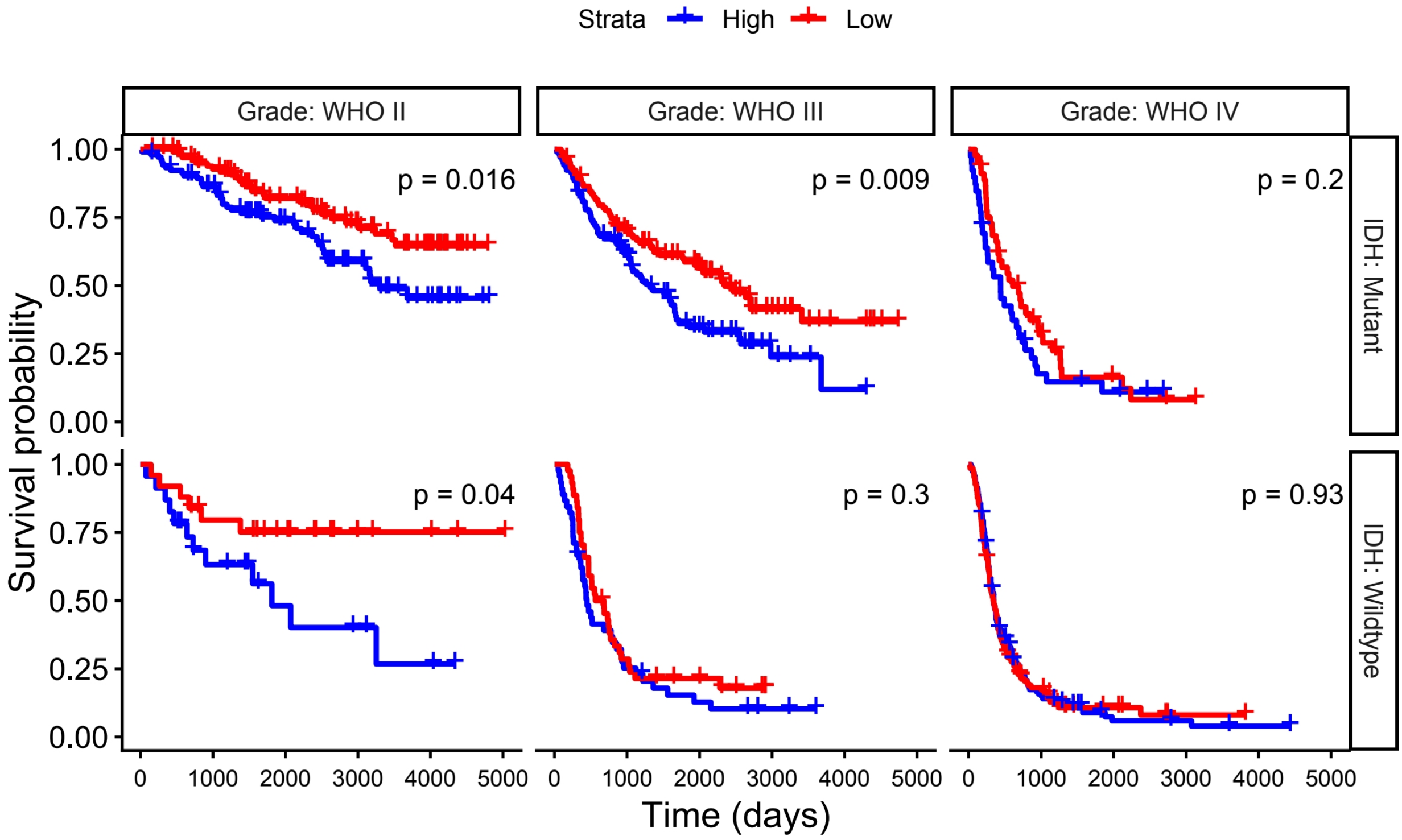

Figure S3. Kaplan–Meier evaluation of the CGGA SLC25A28-plus-age score within six groups defined by IDH status and WHO grade. Each subgroup used its own risk-score median with fixed model coefficients. Blue and red indicate high and low risk. Log-rank P values were 0.016, 0.009 and 0.20 in IDH-mutant grades II, III and IV, and 0.040, 0.30 and 0.93 in IDH-wild-type grades II, III and IV, respectively. Time is shown in days. Curves describe within-subgroup score discrimination.

Figure S4

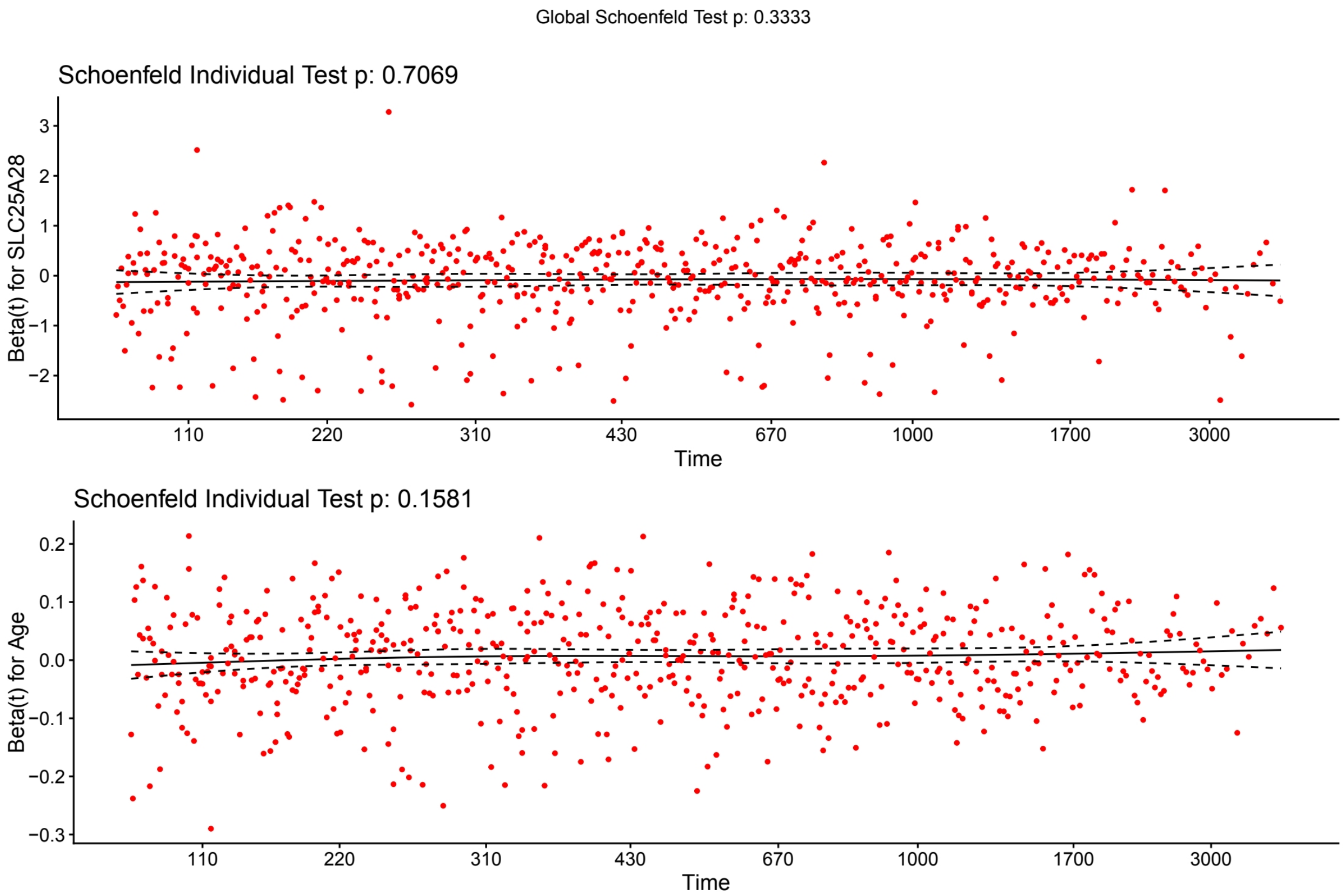

Figure S4. Schoenfeld-residual diagnostics for the CGGA model containing SLC25A28 expression and age. Panels show estimated time-varying coefficients. Individual test P values were 0.7069 and 0.1581, respectively, and the global test P value was 0.3333. The displayed diagnostics evaluate these two model covariates.

Figure S5A

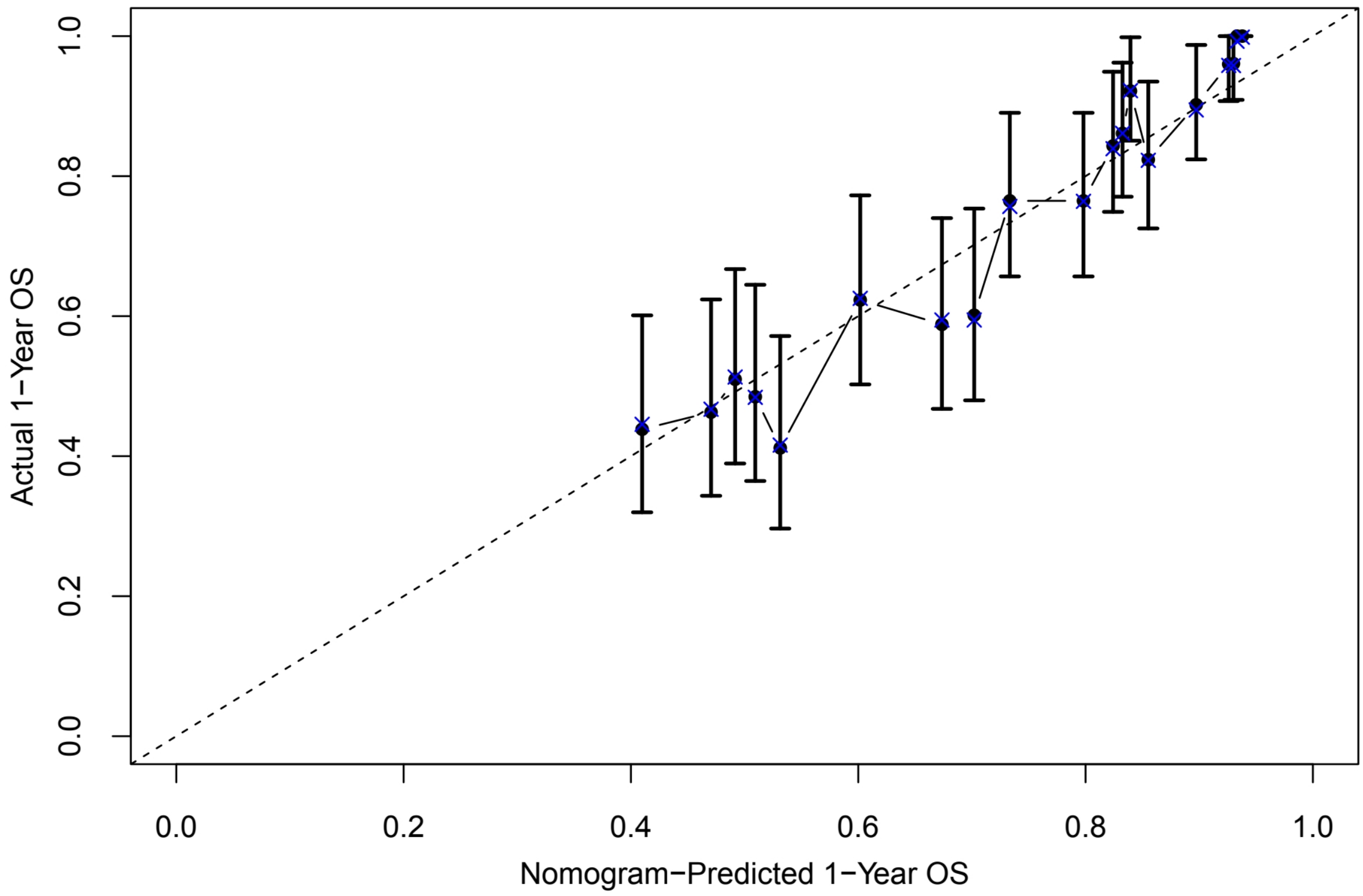

Figure S5A. One-year calibration. Apparent calibration of the CGGA prognostic model in its development sample. Panels A–C compare predicted and observed overall-survival probabilities at 1, 3 and 5 years. The diagonal represents agreement between predicted and observed probabilities. Evaluation used the model-development population.

Figure S5B

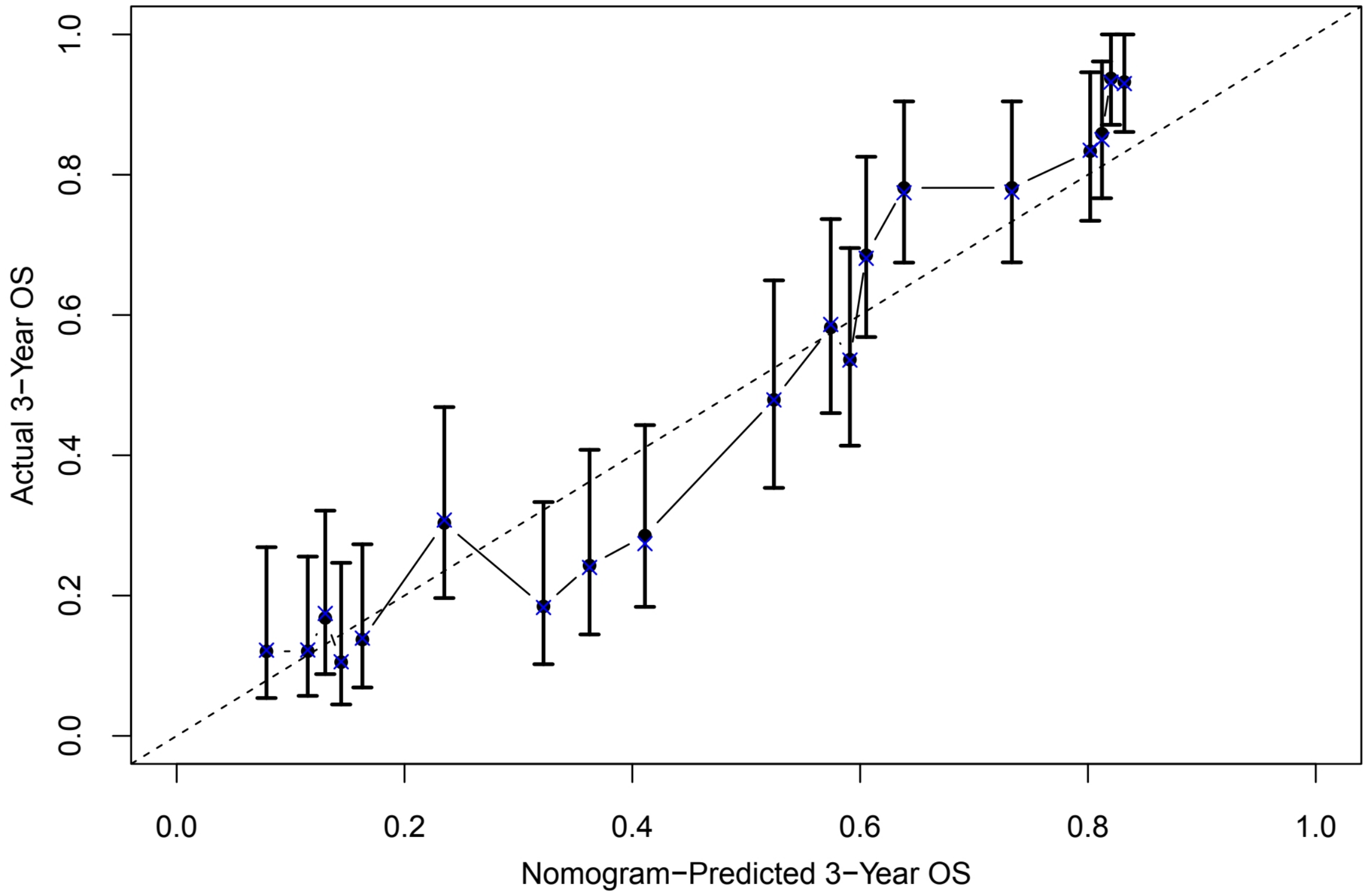

Figure S5B. Three-year calibration. Apparent calibration of the CGGA prognostic model in its development sample. Panels A–C compare predicted and observed overall-survival probabilities at 1, 3 and 5 years. The diagonal represents agreement between predicted and observed probabilities. Evaluation used the model-development population.

Figure S5C

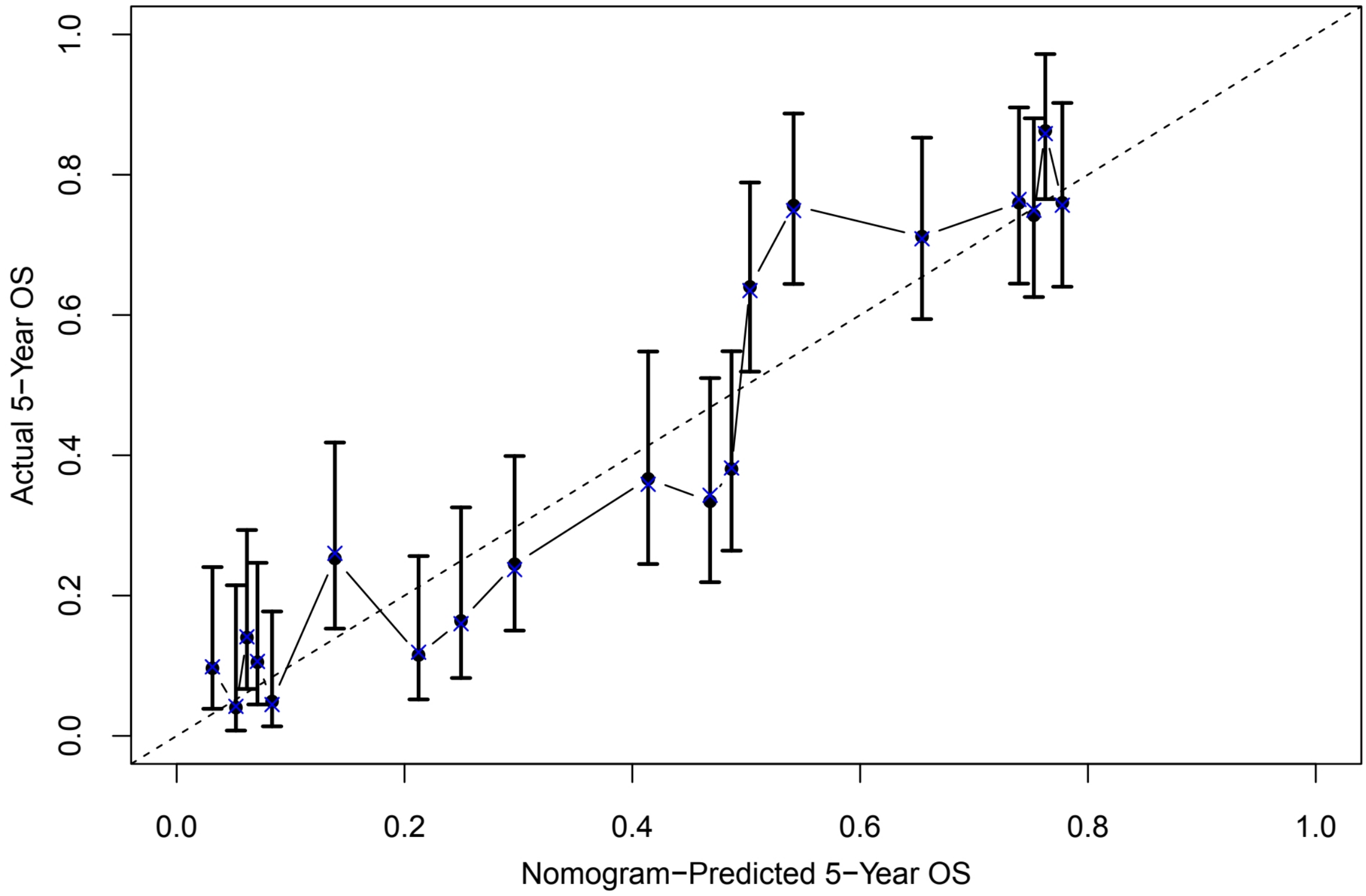

Figure S5C. Five-year calibration. Apparent calibration of the CGGA prognostic model in its development sample. Panels A–C compare predicted and observed overall-survival probabilities at 1, 3 and 5 years. The diagonal represents agreement between predicted and observed probabilities. Evaluation used the model-development population.

Figure S6A

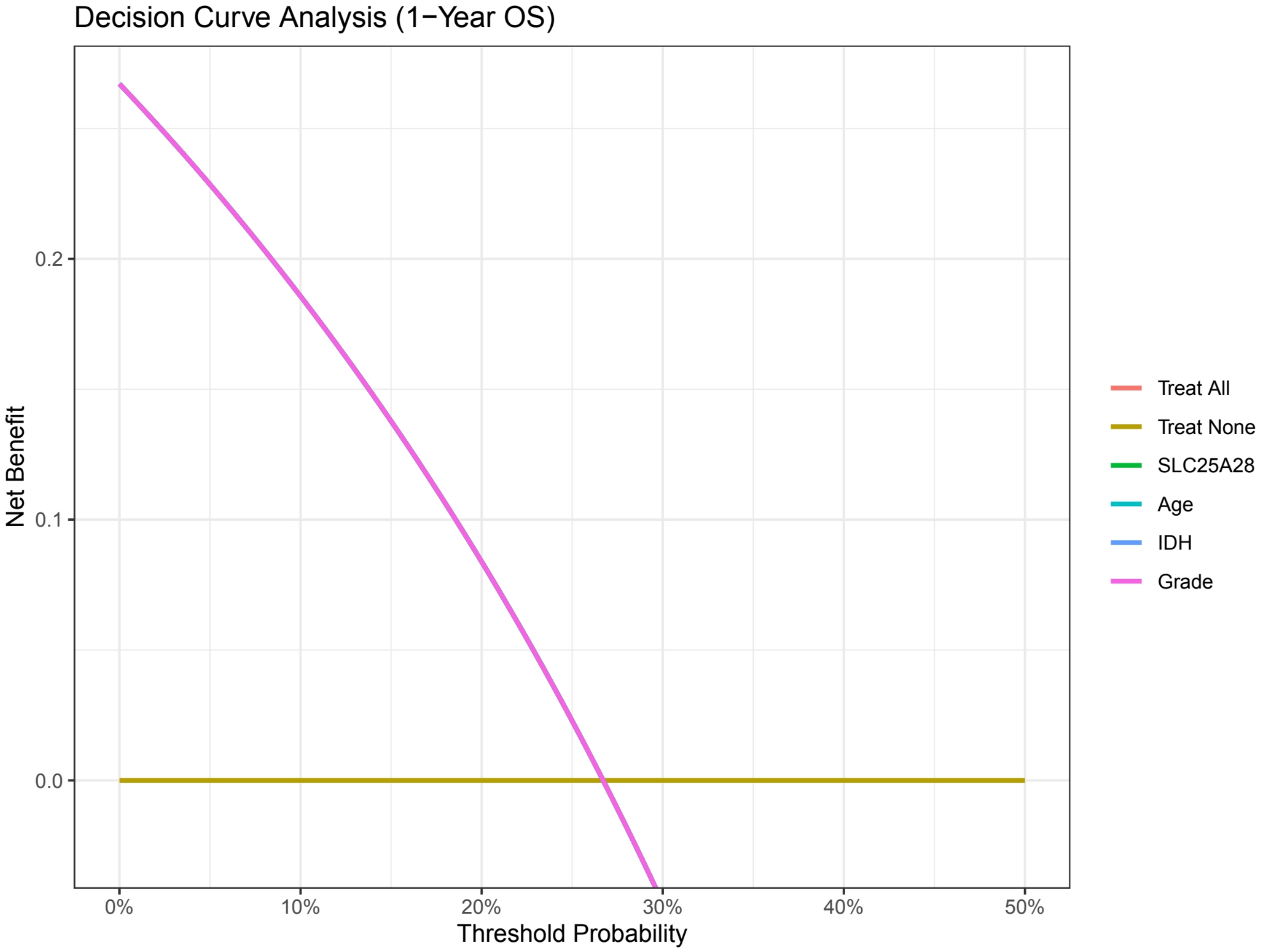

Figure S6A. 1-year decision curves in CGGA. Exploratory decision curves for individual predictors in CGGA and TCGA. Curves are shown at 1 year (A,B), 3 years (C,D), and 5 years (E,F), with CGGA in A, C and E and TCGA in B, D and F. Each colored curve corresponds to a single-predictor assessment of SLC25A28, age, IDH status or WHO grade; treat-all and treat-none strategies provide reference curves. The horizontal axis denotes threshold probability and the vertical axis net benefit. The curves evaluate single predictors, separately from the combined prognostic score.

Figure S6B

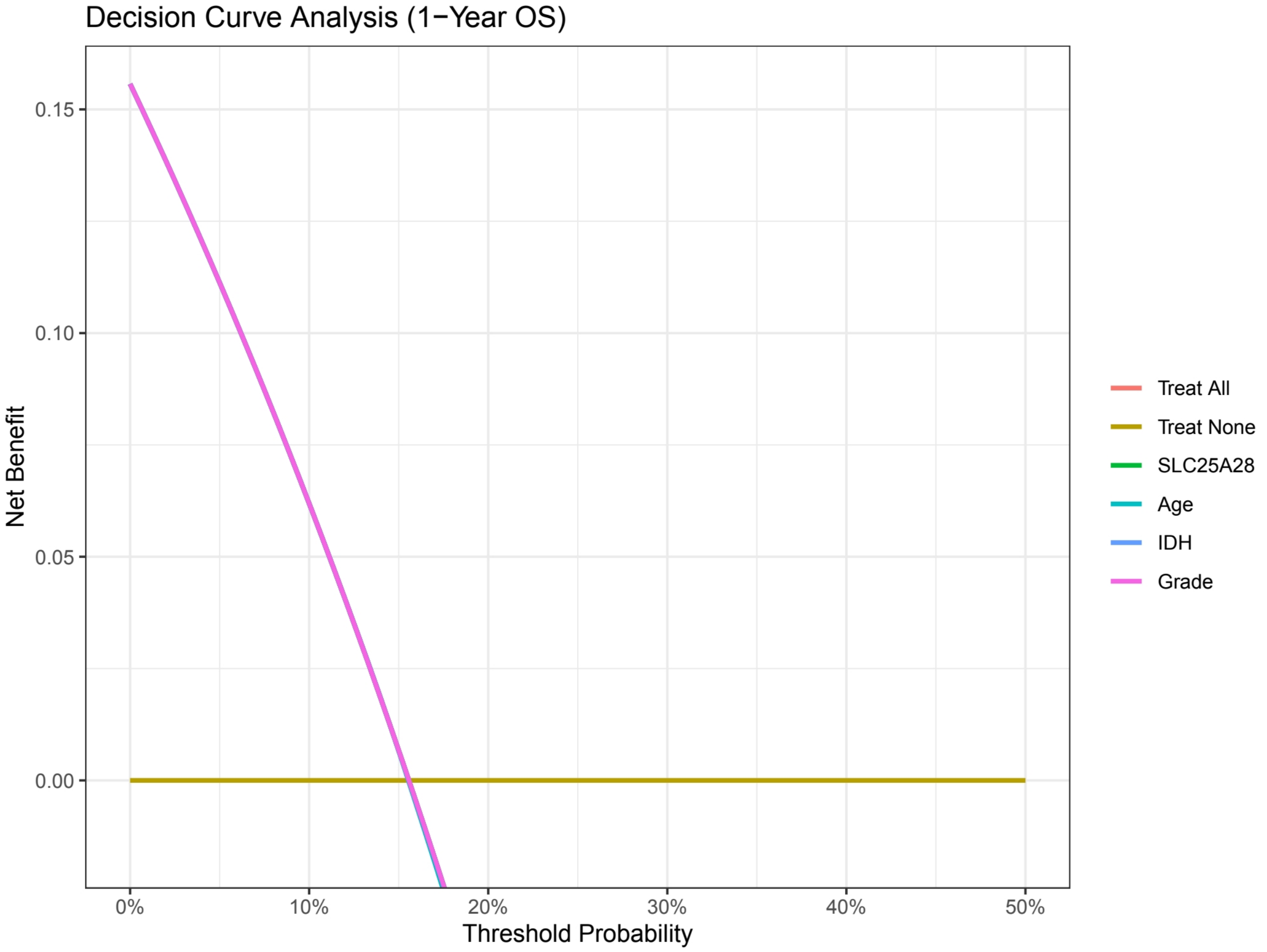

Figure S6B. 1-year decision curves in TCGA. Exploratory decision curves for individual predictors in CGGA and TCGA. Curves are shown at 1 year (A,B), 3 years (C,D), and 5 years (E,F), with CGGA in A, C and E and TCGA in B, D and F. Each colored curve corresponds to a single-predictor assessment of SLC25A28, age, IDH status or WHO grade; treat-all and treat-none strategies provide reference curves. The horizontal axis denotes threshold probability and the vertical axis net benefit. The curves evaluate single predictors, separately from the combined prognostic score.

Figure S6C

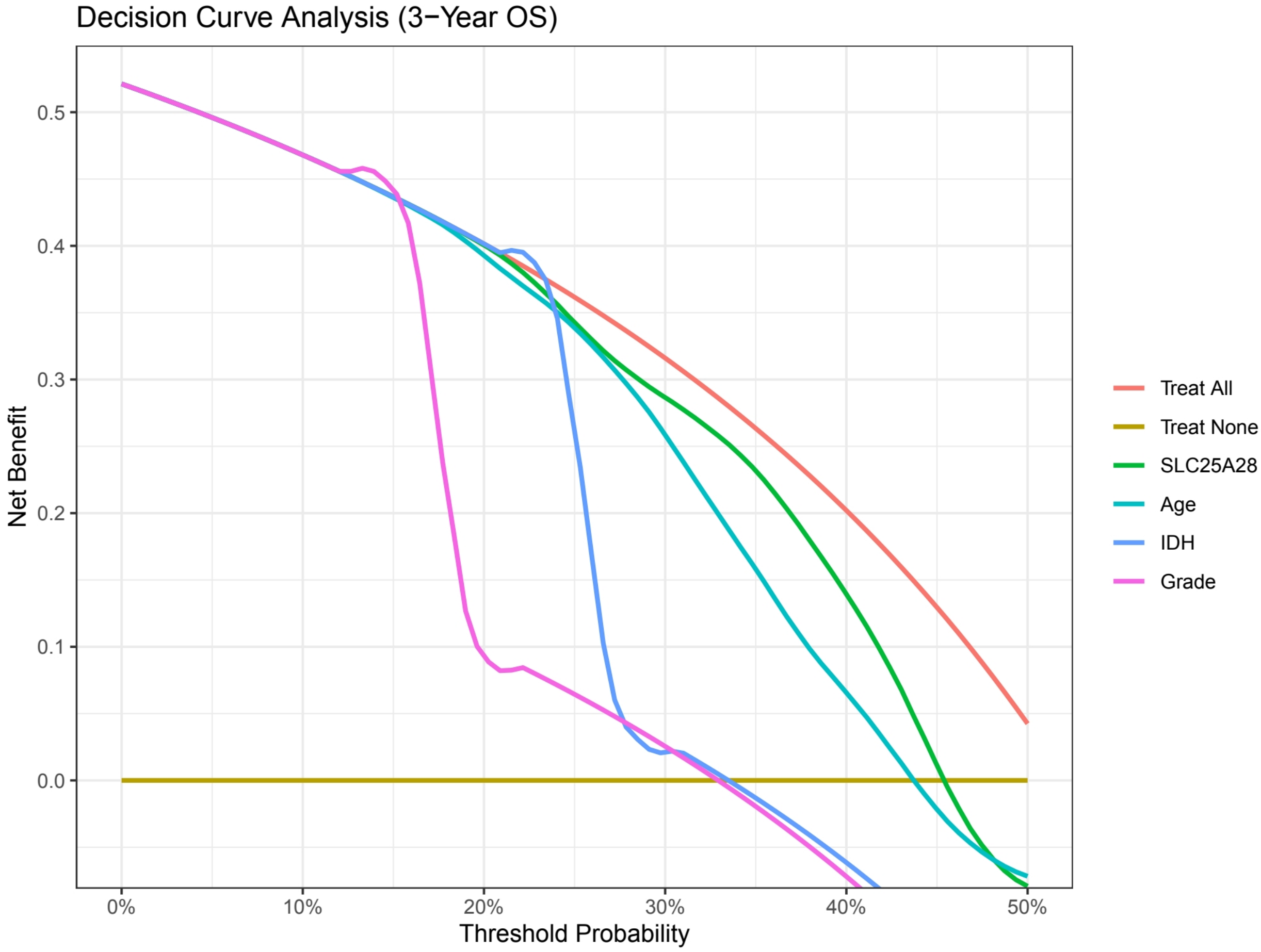

Figure S6C. 3-year decision curves in CGGA. Exploratory decision curves for individual predictors in CGGA and TCGA. Curves are shown at 1 year (A,B), 3 years (C,D), and 5 years (E,F), with CGGA in A, C and E and TCGA in B, D and F. Each colored curve corresponds to a single-predictor assessment of SLC25A28, age, IDH status or WHO grade; treat-all and treat-none strategies provide reference curves. The horizontal axis denotes threshold probability and the vertical axis net benefit. The curves evaluate single predictors, separately from the combined prognostic score.

Figure S6D

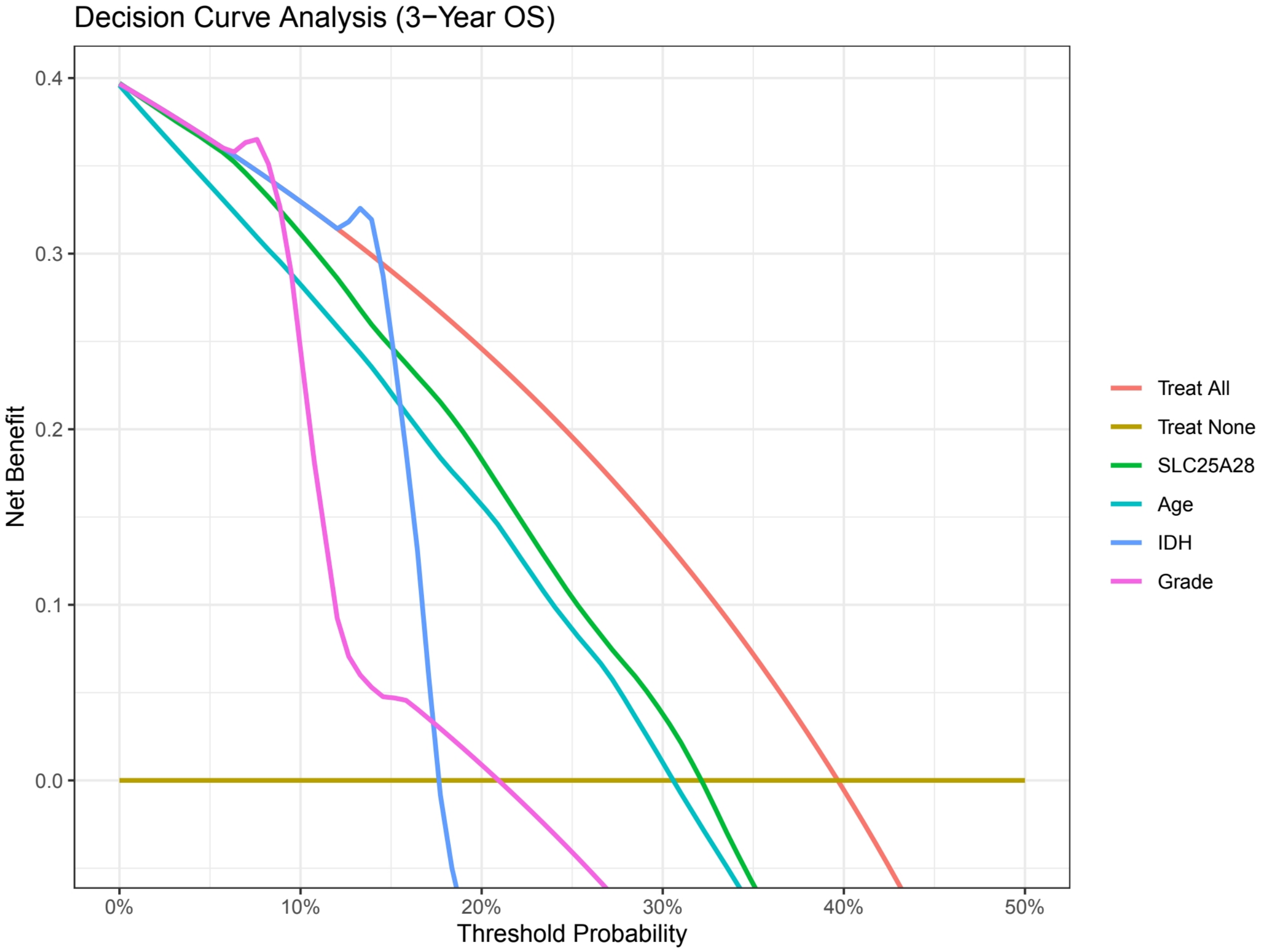

Figure S6D. 3-year decision curves in TCGA. Exploratory decision curves for individual predictors in CGGA and TCGA. Curves are shown at 1 year (A,B), 3 years (C,D), and 5 years (E,F), with CGGA in A, C and E and TCGA in B, D and F. Each colored curve corresponds to a single-predictor assessment of SLC25A28, age, IDH status or WHO grade; treat-all and treat-none strategies provide reference curves. The horizontal axis denotes threshold probability and the vertical axis net benefit. The curves evaluate single predictors, separately from the combined prognostic score.

Figure S6E

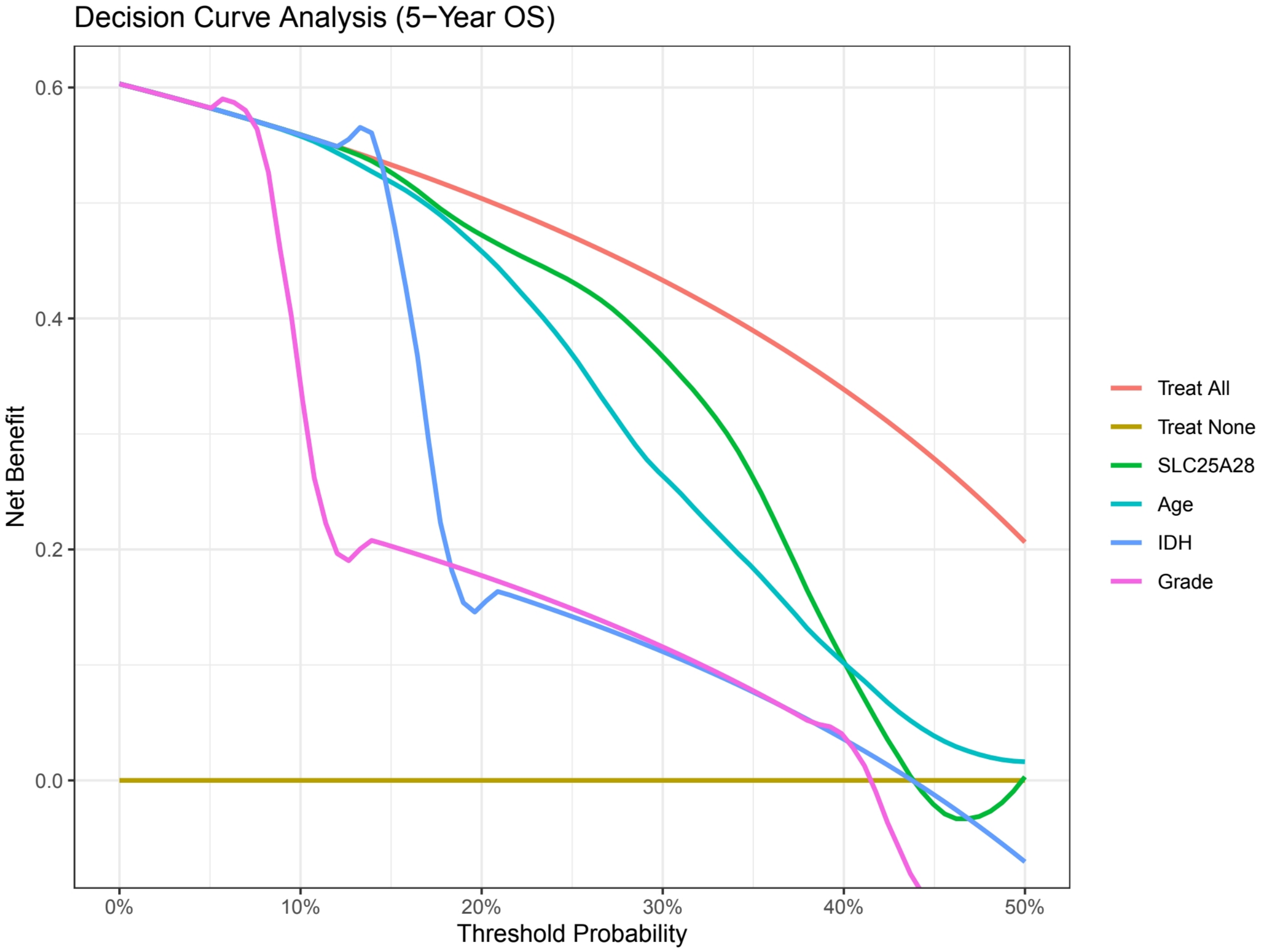

Figure S6E. 5-year decision curves in CGGA. Exploratory decision curves for individual predictors in CGGA and TCGA. Curves are shown at 1 year (A,B), 3 years (C,D), and 5 years (E,F), with CGGA in A, C and E and TCGA in B, D and F. Each colored curve corresponds to a single-predictor assessment of SLC25A28, age, IDH status or WHO grade; treat-all and treat-none strategies provide reference curves. The horizontal axis denotes threshold probability and the vertical axis net benefit. The curves evaluate single predictors, separately from the combined prognostic score.

Figure S6F

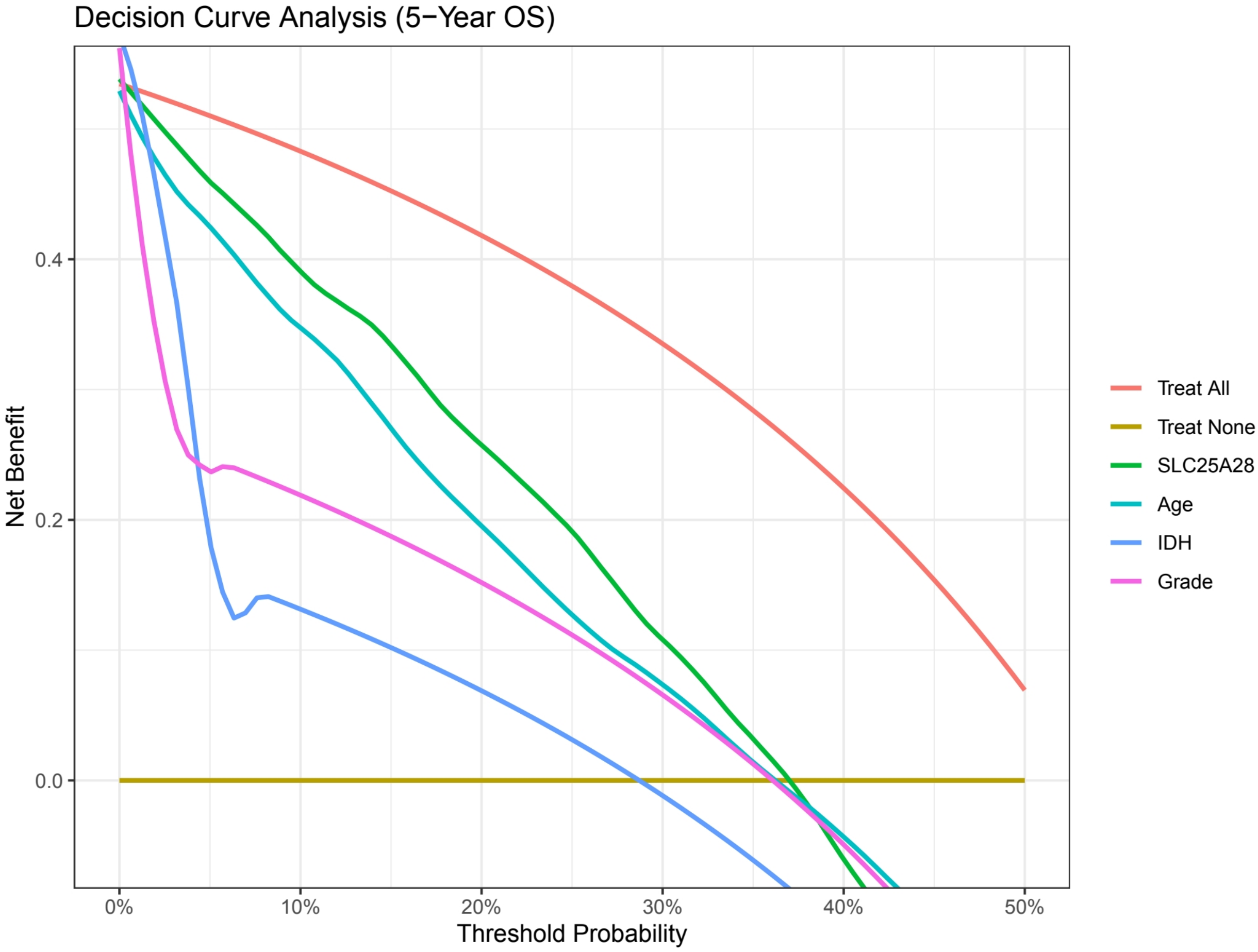

Figure S6F. 5-year decision curves in TCGA. Exploratory decision curves for individual predictors in CGGA and TCGA. Curves are shown at 1 year (A,B), 3 years (C,D), and 5 years (E,F), with CGGA in A, C and E and TCGA in B, D and F. Each colored curve corresponds to a single-predictor assessment of SLC25A28, age, IDH status or WHO grade; treat-all and treat-none strategies provide reference curves. The horizontal axis denotes threshold probability and the vertical axis net benefit. The curves evaluate single predictors, separately from the combined prognostic score.

Figure S7

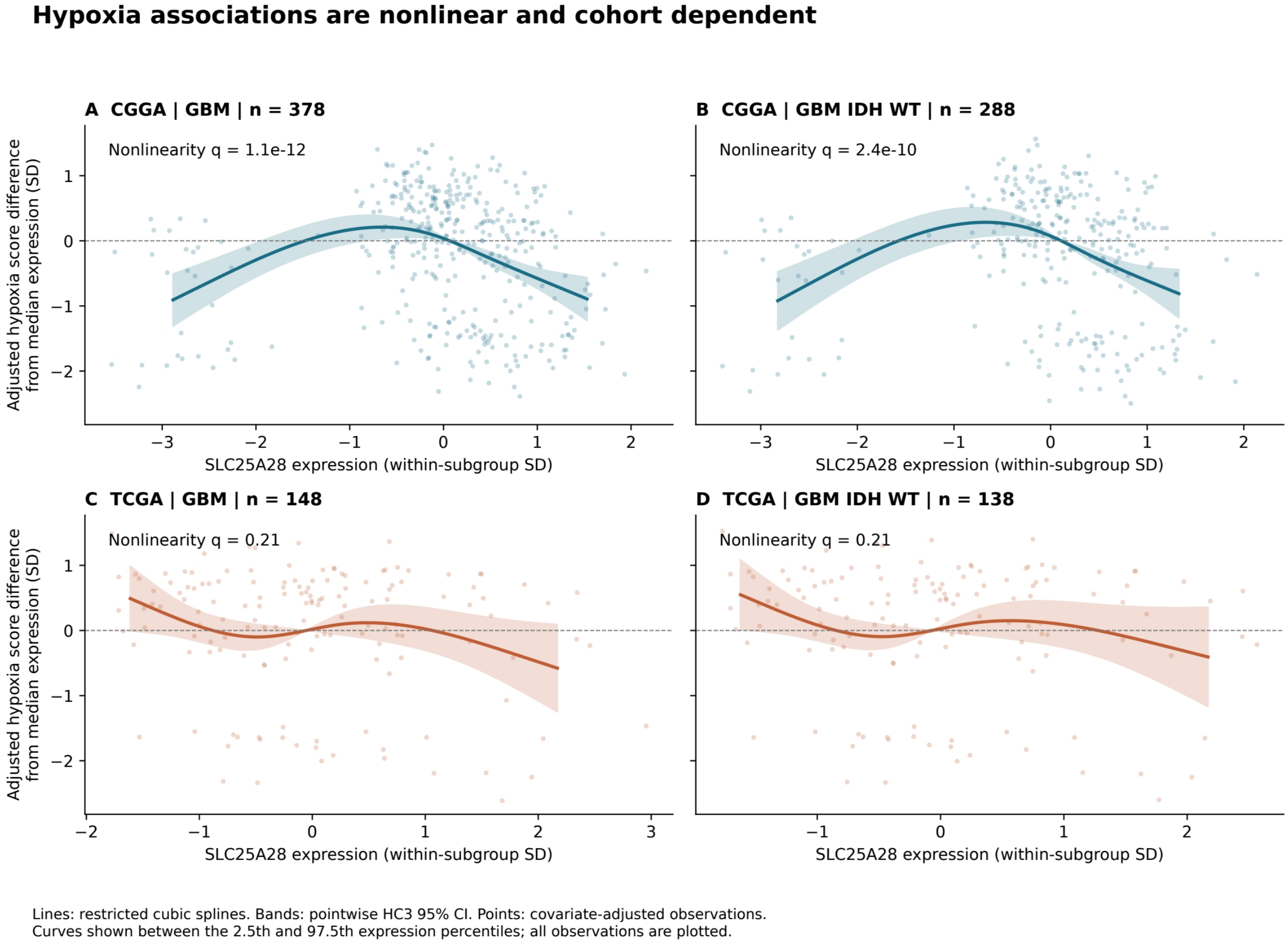

Figure S7. Nonlinear associations between SLC25A28 expression and Hallmark hypoxia scores. Restricted cubic splines are shown for CGGA historical GBM (A), CGGA GBM/IDH-wild-type (B), TCGA historical GBM (C) and TCGA GBM/IDH-wild-type (D). Knots were placed at subgroup expression percentiles 5, 35, 65 and 95. Models used the extended covariates described for Figure 10. The horizontal axis is standardized expression; the vertical axis is the fitted standardized score difference from the value at median expression. Points show observations after removing fitted covariate effects and centering at that reference. Shaded bands are pointwise HC3 95% confidence intervals, not simultaneous confidence bands. Curves are displayed between the 2.5th and 97.5th expression percentiles, with all observations plotted. Nonlinearity q values are BH-corrected across 32 cohort-signature-subgroup tests. Curves are exploratory and do not imply a causal or universally reproducible dose-response relationship.

Figure S8

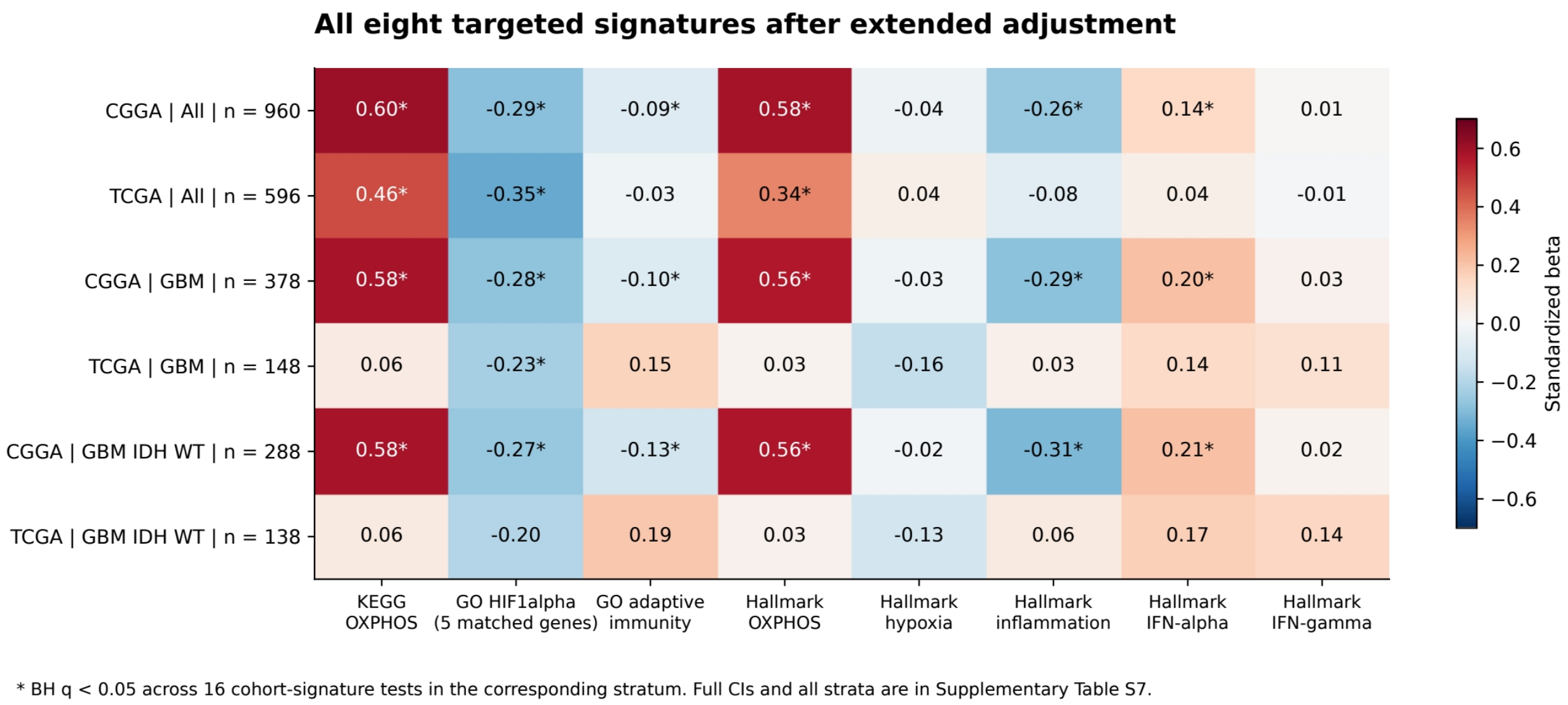

Figure S8. All eight targeted signatures in extended-adjusted models. Cells show standardized continuous-expression coefficients for each signature in the full cohorts, historical GBM and GBM/IDH-wild-type tumors. Models and standardization follow the Methods; full-cohort models include grade, IDH, age, sex and CGGA tumor status. Color denotes coefficient direction and magnitude. Asterisks indicate BH q < 0.05 across 16 cohort-signature tests within the corresponding stratum. The small GO HIF1alpha set includes five matched genes per cohort. Lack of an asterisk does not demonstrate absence of an association. Sample sizes differ from the primary all-grade models in Figure 10 because of additional covariate requirements.

Figure S9

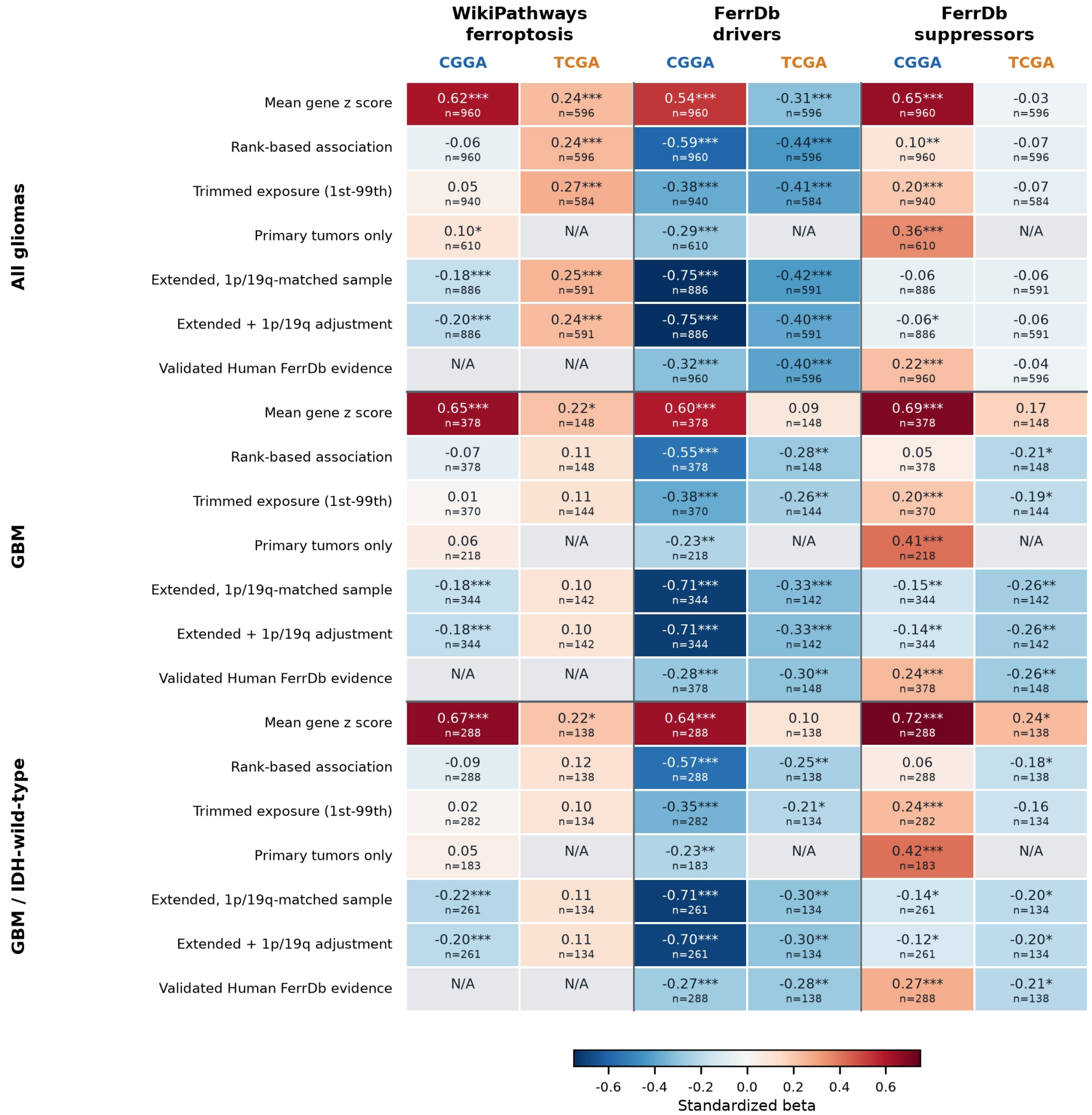

All 111 planned sensitivity tests share one BH-FDR family; every planned estimate is shown.

* q < 0.05; ** q < 0.01; *** q < 0.001. N/A: not planned; NE: not estimable.

Figure S9. Complete sensitivity analyses of ferroptosis-related transcriptional associations. Cells show the standardized SLC25A28 coefficient, its FDR significance indicator, and the number analyzed in extended models. Positive and negative coefficients are shown in red and blue. Rows include mean gene-z-score scoring, rank-transformed exposure and score, removal of exposure values outside the complete-case 1st and 99th percentiles, restriction to primary CGGA tumors, the extended model in samples with known 1p/19q status, additional 1p/19q adjustment in those same samples, and a restricted FerrDb score using genes with at least one Human Validated evidence record. The latter applies only to the driver and suppressor signatures, retaining 267 driver genes and 510 suppressor genes from the primary 300- and 549-gene sets, respectively. All 111 planned sensitivity tests, including the same-sample 1p/19q reference coefficients, share one BH-FDR family; no nonsignificant or directionally discordant estimate is removed. N/A denotes a combination not planned; NE denotes a planned but nonestimable model. Asterisks have the same thresholds as Figure 11. The three strata are All gliomas, histological GBM, and GBM/IDH-wild-type. These analyses assess sensitivity of transcriptional associations, not ferroptosis activity.

Figure S10

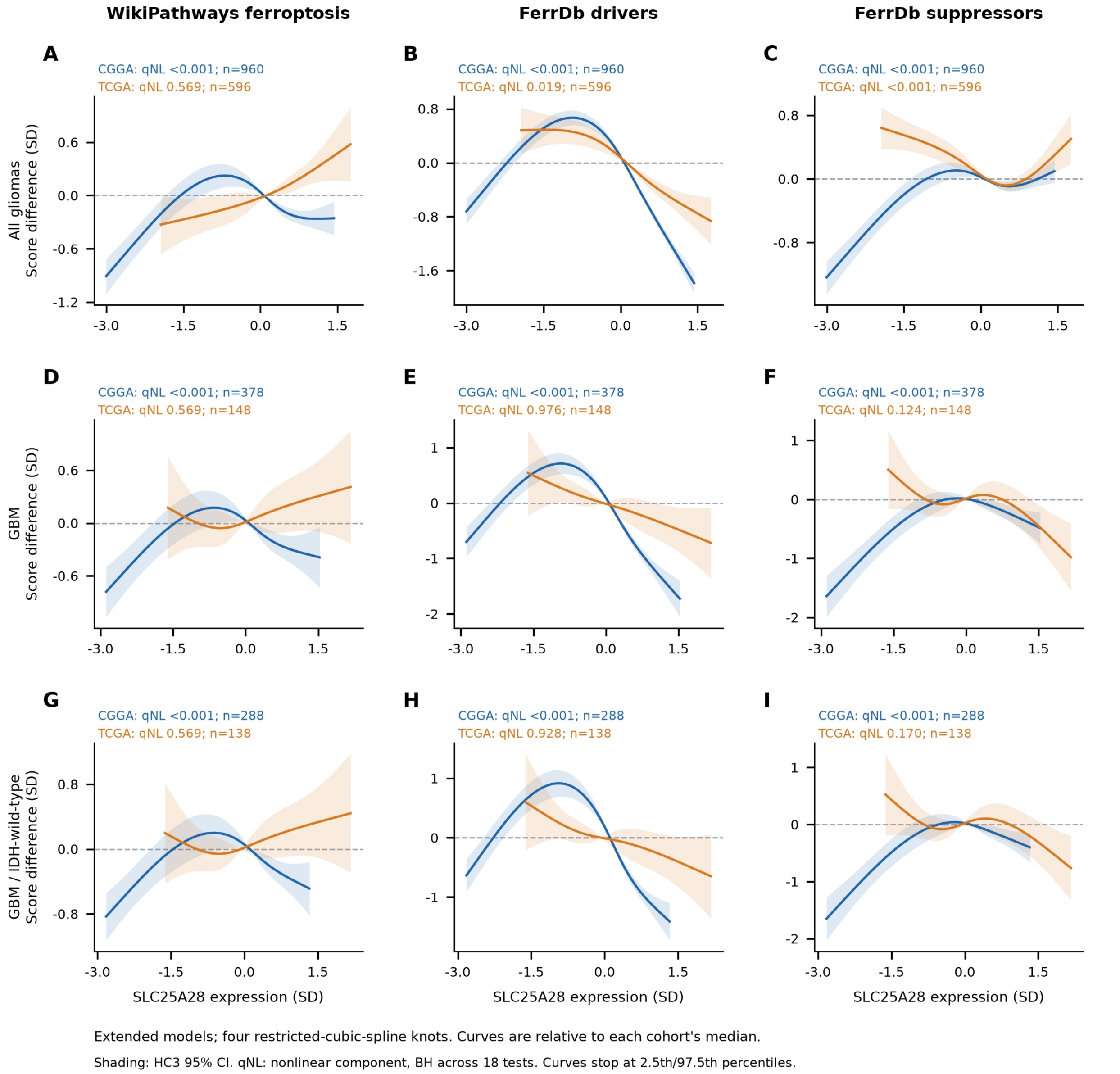

Figure S10. Nonlinear associations between SLC25A28 and ferroptosis-related signatures. Rows correspond to All gliomas, histological GBM, and GBM/IDH-wild-type; columns correspond to the WikiPathways ferroptosis, FerrDb driver, and FerrDb suppressor signatures. Extended models use four knots at the 5th, 35th, 65th, and 95th percentiles of within-analysis SLC25A28 expression. Curves show the model-estimated score difference, in within-analysis standard-deviation units, relative to each cohort's median SLC25A28 value. Blue and orange indicate CGGA and TCGA; shading represents HC3 95% confidence intervals. Each curve is restricted to its cohort-specific 2.5th-97.5th percentile range, without extrapolation. The x axis is standardized separately within each analysis population. qNL is the BH-adjusted robust test of the two nonlinear spline components across all 18 planned nonlinear tests; the distinct family of 18 overall spline tests is reported in the statistical table. n denotes the analyzed sample count. These curves describe transcriptional associations and cannot establish changes in ferroptosis activity.

Figure S11

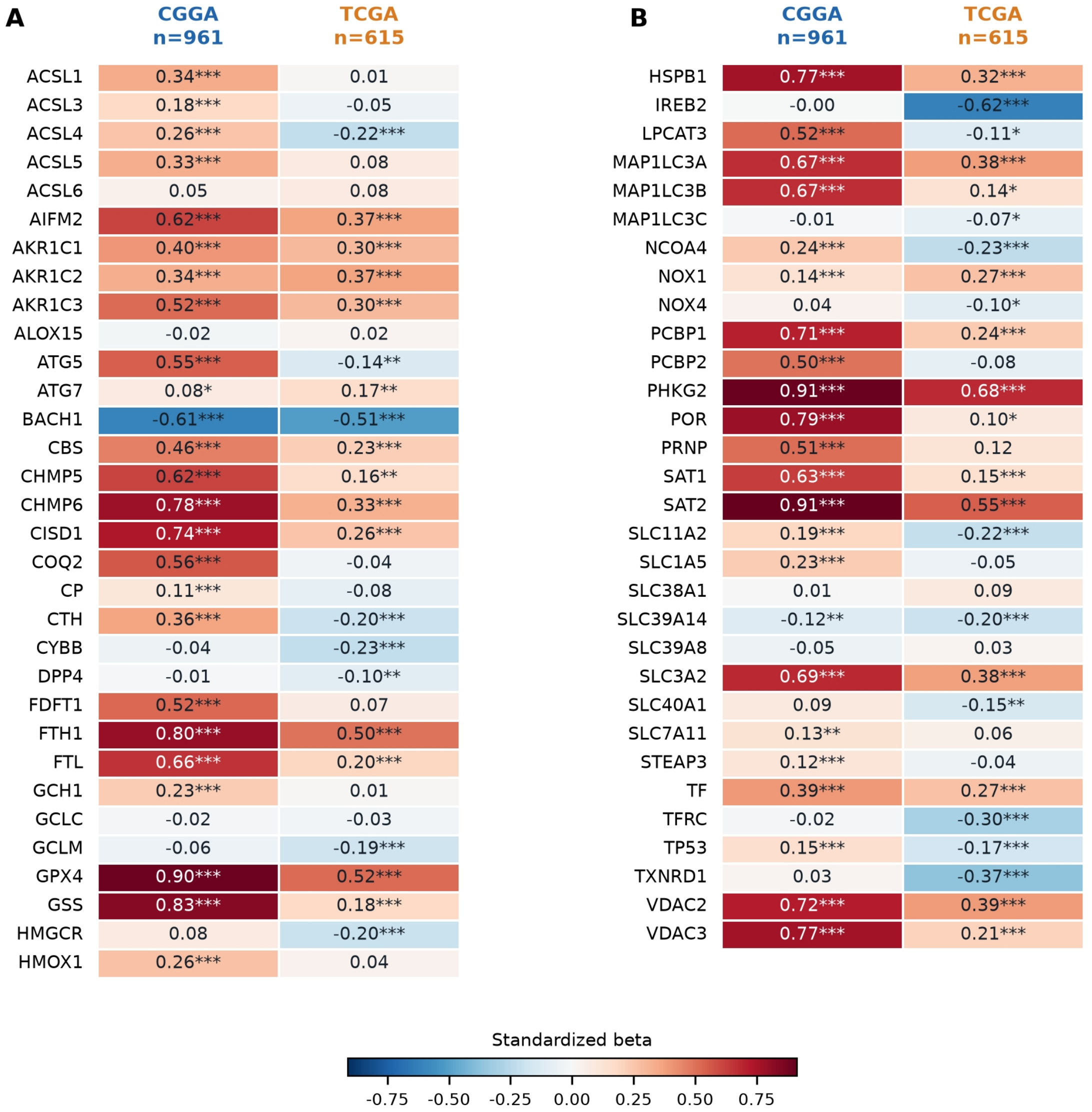

All 63 genes; Grade + IDH adjustment; BH-FDR across all 126 gene-cohort tests.
* q < 0.05; ** q < 0.01; *** q < 0.001. Gene order: down A, then down B.

Figure S11. Gene-level associations within the WikiPathways ferroptosis set. All 63 matched genes in the scoring set are displayed in alphabetical order across panels A (first 32 genes) and B (remaining 31 genes). Cells show the standardized gene-expression coefficient for SLC25A28 after WHO grade and IDH adjustment, with HC3 inference. The BH-FDR correction spans all 126 gene-cohort tests. Positive and negative coefficients are shown in red and blue; asterisks have the same thresholds as Figure 11. Sample sizes are given in the cohort headings. This exploratory decomposition uses the same transcriptomic data as the signature analyses and is not independent validation. Gene-level coexpression and the pathway score do not establish ferroptosis activity or a causal regulatory effect.
